# DNAJC5 facilitates USP19-dependent unconventional secretion of misfolded cytosolic proteins

**DOI:** 10.1038/s41421-018-0012-7

**Published:** 2018-03-06

**Authors:** Yue Xu, Lei Cui, Anthony Dibello, Lihui Wang, Juhyung Lee, Layla Saidi, Jin-Gu Lee, Yihong Ye

**Affiliations:** Laboratory of Molecular Biology, National Institute of Diabetes and Digestive and Kidney Diseases, National Institutes of Health, Bethesda, MD 20892 USA

## Abstract

Cell-to-cell transmission of misfolded proteins propagates proteotoxic stress in multicellular organisms when transmitted polypeptides serve as a seeding template to cause protein misfolding in recipient cells, but how misfolded proteins are released from cells to initiate this process is unclear. Misfolding-associated protein secretion (MAPS) is an unconventional protein-disposing mechanism that specifically exports misfolded cytosolic proteins including various neurodegenerative disease-causing proteins. Here we establish the HSC70 co-chaperone DNAJC5 as an essential mediator of MAPS. USP19, a previously uncovered MAPS regulator binds HSC70 and acts upstream of HSC70 and DNAJC5. We further show that as a membrane-associated protein localized preferentially to late endosomes and lysosomes, DNAJC5 can chaperone MAPS client proteins to the cell exterior. Intriguingly, upon secretion, misfolded proteins can be taken up through endocytosis and eventually degraded in the lysosome. Collectively, these findings suggest a transcellular protein quality control regulatory pathway in which a deubiquitinase-chaperone axis forms a “triaging hub”, transferring aberrant polypeptides from stressed cells to healthy ones for disposal.

## Introduction

Misfolded proteins pose a major threat to the protein homeostasis network of eukaryotic cells, particularly in terminally differentiated cells like neurons, which cannot proliferate to dilute aberrant polypeptides to a level below aggregation threshold. To safeguard the protein homeostasis network, cells have evolved several protein quality control (PQC) strategies including chaperone-assisted protein folding, proteasomal degradation, and autophagy-mediated protein turnover^1, 2^. As anticipated, failures in PQC lead to accumulation of aggregation–prone polypeptides, culminating in proteotoxic stress that can cause a variety of human diseases^2^.

Misfolding-associated protein secretion (MAPS) is a recently uncovered PQC mechanism, which targets misfolded cytosolic proteins to an unconventional protein secretion (UPS) pathway for export into the extracellular space^3^. In this process, the endoplasmic reticulum (ER)-associated deubiquitinase USP19 uses an intrinsic chaperone activity to enrich misfolded proteins on the ER surface. Subsequently, misfolded polypeptides are moved into the lumen of a population of ER-associated late endosomes via an unknown protein translocation mechanism. These proteins are eventually secreted when partial or complete membrane fusion occurs between endosomes and the plasma membrane (PM). Interestingly, many MAPS substrates are also ubiquitinated and targeted for degradation by the proteasome in cells^3, 4^. The interaction of USP19 with substrates causes the removal of ubiquitin chains, which facilitates the export of MAPS substrates^3^. Existing evidence suggests that MAPS is a supplementary PQC process in parallel with the proteasome to alleviate proteotoxic stress caused by haplo-insufficiency of the proteasome function^3^.

Although USP19 is known to interact with Hsp90 and Hsp70^5^, the chaperone requirement for MAPS has been unclear. In addition, the pathway has so far only been characterized with a few substrates, and therefore, it is unclear whether MAPS can be used to export most misfolded cytosolic proteins. Moreover, among the polypeptides examined, α-synuclein (α-Syn), an intrinsically misfolded soluble protein implicated in Parkinson’s disease is efficiently secreted, whereas the Alzheimer’s disease-associated Tau protein, when fused with a GFP tag was not subject to secretion by MAPS^3^. Thus, it is unclear to what extent MAPS may contribute to the widely reported cell-to-cell transmission of misfolded proteins in neurodegenerative diseases^6, 7^.

In addition to MAPS, several UPS routes have been reported previously^8–10^. A few studies suggested exosome or extracellular vesicles as carriers for misfolded α-Syn and Tau^11–14^. However, our results as well as other studies showed that misfolded α-Syn and Tau released into the cell exterior are mostly not bound to any vesicle^3, 15, 16^. Thus, the relative contribution of different protein secretion routes to intercellular propagation of misfolded neurotoxic proteins under physiological conditions needs to be further evaluated. Interestingly, a recent study suggested a mechanism reminiscent of MAPS for disposal of Tau and α-Syn, which involves the cytosolic chaperone HSC70, its co-chaperone DNAJC5 and a vesicle fusion regulator SNAP23^15^. However, the functional relationship between these processes is unclear.

In this study, we identify additional MAPS substrates, which now cover a collection of aberrant proteins associated with neurodegenerative diseases. Importantly, we characterize the role of two USP19-interacting chaperones; while Hsp90 is dispensable for MAPS, HSC70 and its co-chaperone DNAJC5 function together with USP19 to form a critical protein-triaging hub in MAPS. These findings unify two UPS routes that were previously deemed unrelated. Importantly, our study suggests that MAPS, as an exosome-independent secretion process, may contribute to the export of diverse neurotoxic misfolded proteins known to propagate during neurodegeneration.

## Results

### USP19 promotes secretion of disease-causing aberrant proteins

**Fig. 1:**
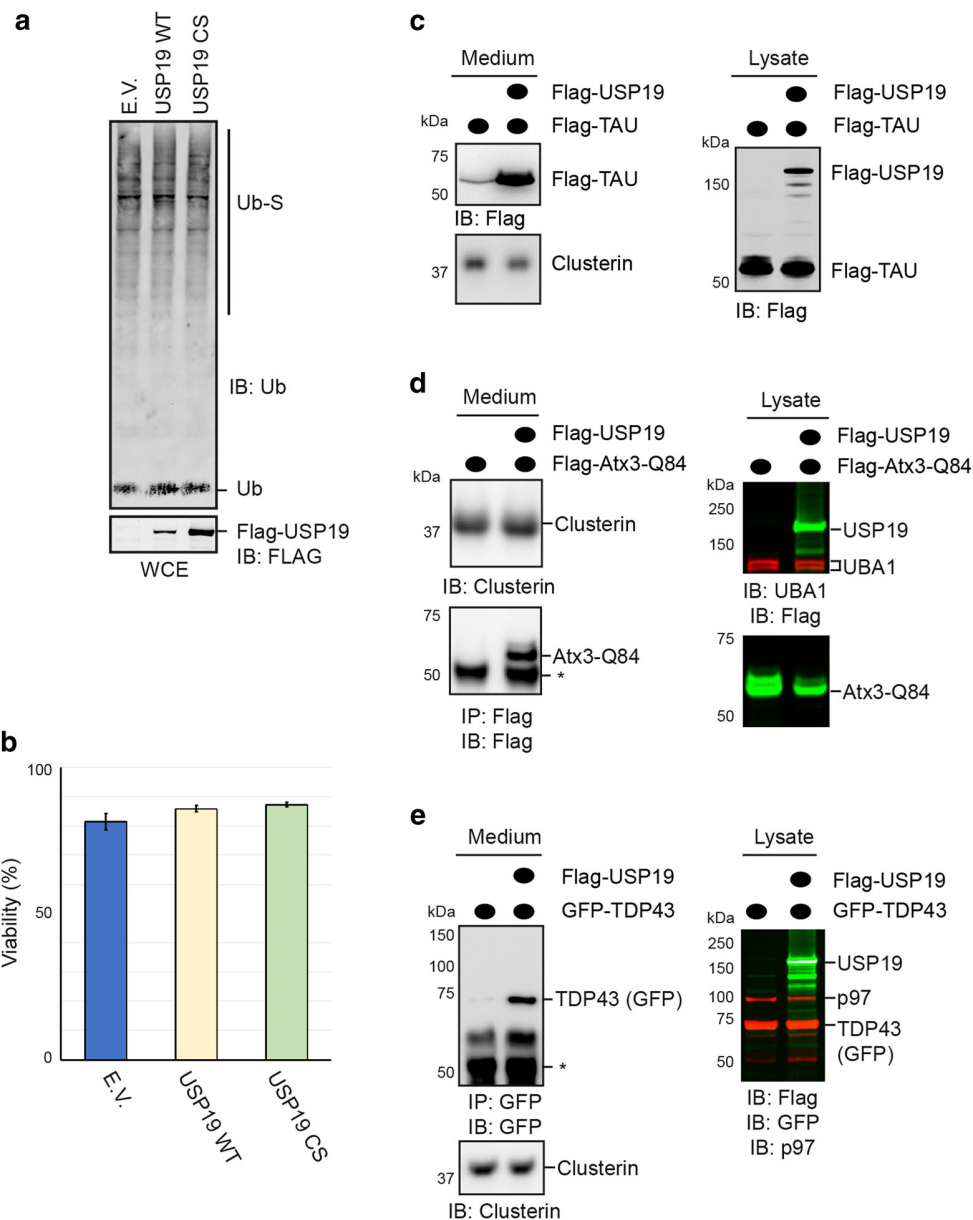
USP19 promotes the secretion of neurotoxic proteins. **a** USP19 overexpression did not alter polyubiquitination profile. Whole cell extracts (WCE) from cells transfected with either an empty vector (E.V.), or wild type (WT) USP19, or USP19 C506S (CS) plasmids for 48 h were analyzed by immunoblotting (IB) Ub-S, ubiquitinated substrates. **b** USP19 overexpression does not affect cell viability. The viability of cells transfected with the indicated plasmids was monitored 48 h post-transfection (mean ± s.e.m., *n* = 3). **c** USP19 stimulates Tau secretion. Conditioned medium and cell lysate from 293T cells transfected as indicated were analyzed by immunoblotting (IB). Note that the secretion of the conventional ER cargo Clusterin is not affected by USP19 overexpression. **d** USP19 promotes the secretion of Atx3-Q84. As in **c**, except that Flag-Atx3-Q84 was used and that the medium was subject to immunoprecipitation (IP) by a Flag antibody followed by immunoblotting. Asterisk, IgG. **e** USP19 promotes the secretion of TDP-43. As in **d**, except that GFP-TDP-43 was tested. Asterisk, IgG

To see whether USP19 is generally involved in secretion of folding defective polypeptides, we tested a collection of wild type (WT) cytosolic proteins that are prone to misfolding. In many cases, mutations in genes encoding these proteins have been linked to neurodegenerative diseases. The client proteins tested include Tau, a microtubule binding protein implicated in Alzheimer’s disease^17^, poly-glutamine-containing ataxin3 known to be involved in spinocerebellar ataxia 3 (SCA3)^18^, amyotrophic lateral sclerosis-associated SOD1^19^ and TDP43^20, 21^. To facilitate the detection of these proteins, they were expressed transiently as tagged proteins in HEK293T cells in the absence or presence of Flag-USP19. Despite overexpression, USP19 did not cause significant change in the global profile of polyubiquitinated proteins. This is also the case for the catalytically inactive USP19 mutant (C506S) (Fig. 1a), suggesting that the activity of USP19 is either tightly regulated or acts only on a small fraction of polyubiquitinated proteins. Importantly, overexpression of either WT USP19 or USP19 C506S did not alter cell viability^3^ even in cells overexpressing a misfolded metastable protein (Fig. 1b). However, under this condition, wild type (WT) USP19 is able to promote the secretion of misfolded α-Synuclein (α-Syn) in a dose-dependent manner (Supplementary Figure 1A), as demonstrated previously^3^. Under this condition, immunoblotting analyses revealed a small amount of Tau and SOD1 in media in the absence of USP19 overexpression (Fig. 1c; Supplementary Figure 1B). By contrast, for ataxin3-Q84 and TDP43, barely any protein was detected in media even after enrichment by immunoprecipitation (Fig. 1d, e). However, when USP19 was overexpressed, the secretion of all four proteins was enhanced (Fig. 1c–e; Supplementary Figure 1B). Noticeably, unlike Tau and SOD1, the detection of Atx3-Q84 and TDP43 secretion from USP19-overexpressing cells required enrichment by immunoprecipitation (IP) (Fig. 1d, e). Thus, all substrates tested are secreted in a USP19-dependent manner, whereas the secretion of the ER-dependent cargo Clusterin was not affected by USP19 (Fig. 1 c–e).

Because misfolded proteins are prone to aggregation, a factor that might influence the MAPS efficiency is the aggregation propensity, assuming that the MAPS pathway might favor more soluble proteins. To test this idea, we analyzed the secretion of GFP bearing a poly-glutamine tract containing either 25 or 103 glutamine residues. As reported previously^22^, Q25-GFP was soluble, but most cells expressing GFP-Q103 contained one large Q103-GFP-containing puncta, presumably due to protein aggregation (Supplementary Figure 1C). Immunoblotting analysis of conditioned media showed that secretion was readily detected in media collected from cells overexpressing USP19 and GFP-Q25, but in cells expressing GFP-Q103, secretion was not detected regardless of USP19 overexpression (Supplementary Figure 1D). These data suggest that USP19-dependent secretion might preferentially export soluble misfolded proteins or small protein aggregates.

### HSP90 is dispensable for USP19-mediated MAPS

**Fig. 2:**
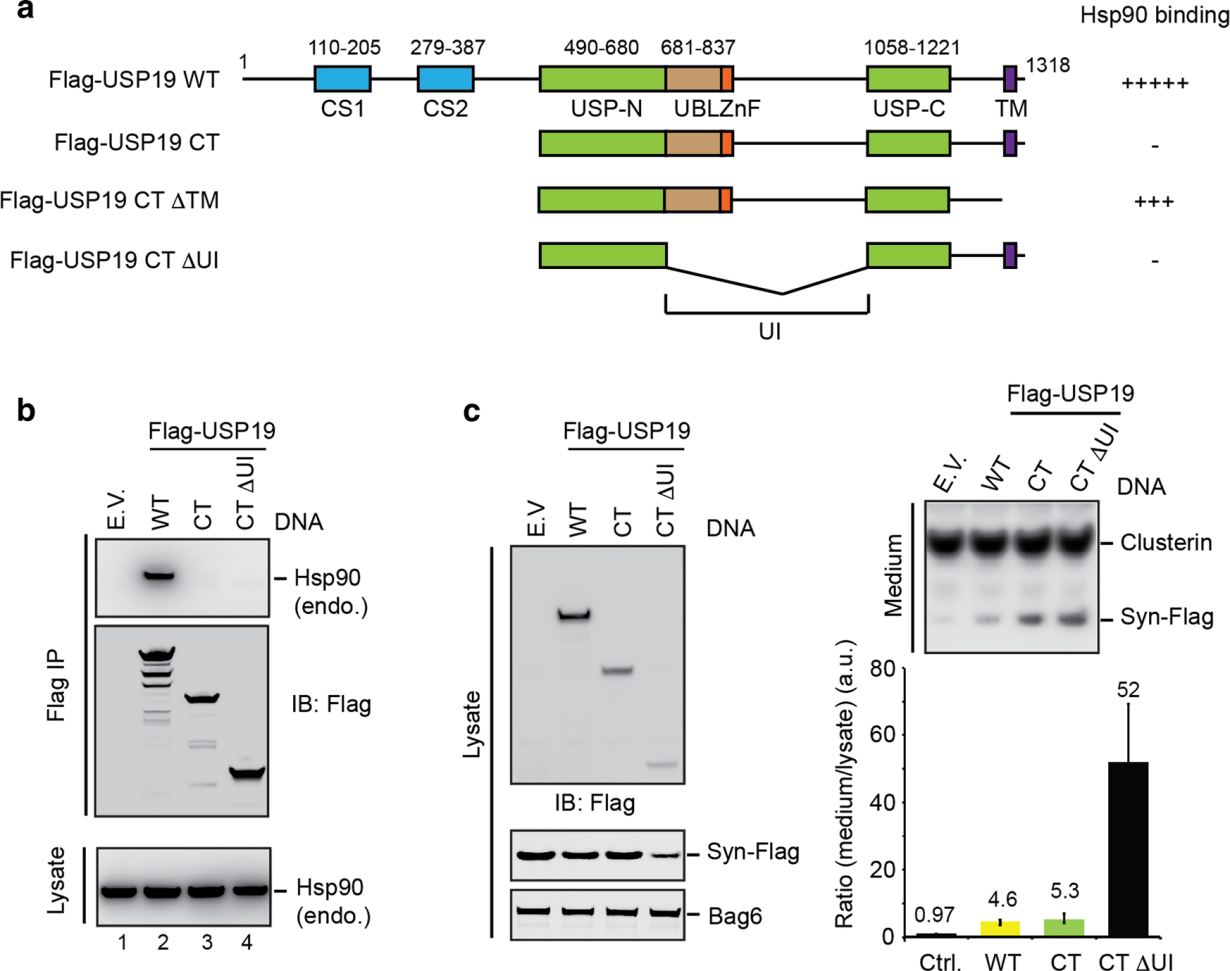
Hsp90 interaction is dispensable for USP19-stimulated secretion. **a** A schematic illustration of the USP19 constructs used in the study. UI UBL-containing insertion. **b** Cell lysates prepared from 293T cells expressing the indicated USP19 variants were subject to immunoprecipitation (IP) followed by immunoblotting. As a negative control, cells were transfected with an empty vector (E.V.). **c** A USP19 mutant lacking the UBL-containing insertion (UI) is a stronger MAPS stimulator. Secretion of α-Syn-Flag from cells expressing the indicated USP19 variants was analyzed by immunoblotting. The graph shows the relative level of α-Syn secretion normalized by α-Syn in cell lysates (mean ± s.e.m., *n* = 3)

We next sought to identify additional MAPS regulators by examining the published USP19 interactome^5^. Because we previously demonstrated a specific interaction between USP19 and the heat shock protein Hsp90^5^, we first tested whether Hsp90 is required for MAPS. To this end, we generated a series of USP19 truncation mutants to define the domain in USP19 required for Hsp90 association (Fig. 2a). We performed co-IP using cells transfected with these USP19 variants. Interestingly, when USP19 has the C-terminal transmembrane domain, its interaction with endogenous Hsp90 was exclusively mediated by an *N*-terminal segment (1–489) (Fig. 2b, lane 3 vs. 2). However, when the TM domain was removed, the C-terminal (CT) fragment of USP19 could now bind Hsp90 almost as efficiently as full-length WT USP19 (Supplementary Figure 2A). This activity was mainly attributed to a ubiquitin-like (UBL) insertion (UI) that divides the USP domain into a *N*- and *C*-terminal half. We conclude that USP19 has two Hsp90 binding sites, but the one present in the USP domain is normally suppressed for membrane-associated USP19 isoforms.

We next tested whether USP19 mutants defective in Hsp90 binding (USP19 CT and USP19 CT ΔUI) could still promote the secretion of MAPS substrates such as α-Syn, unassembled Ubl4A, Tau, and truncated GFP (GFP1-10). We analyzed conditioned media and cell lysate collected from cells transfected with individual MAPS substrate together with these USP19 variants. Secreted proteins were quantified by immunoblotting and the secretion efficiency was normalized using the protein level in lysate as a reference. The results showed that the mutant lacking the *N*-terminal CS domains (USP19 CT) enhanced MAPS similarly as WT USP19 (Fig. 2c). Intriguingly, removal of the UI domain from USP19 CT (USP19 CT ∆UI) dramatically increased its MAPS-stimulating activity, as demonstrated using α-Syn (Fig. 2c) and Ubl4A as substrate (Supplementary Figure 2B, C). USP19 CT ∆UI also stimulated GFP1-10 and Tau secretion (Supplementary Figure 2D, E). These observations indicate that the ability to associate with Hsp90 is dispensable for USP19-stimulated MAPS. Furthermore, it appears that the UI domain has a regulatory role that normally suppresses MAPS.

To further explore the relationship between Hsp90 and MAPS, we treated cells expressing MAPS substrates with Genetespib, a potent Hsp90 inhibitor^23^. After medium change and drug treatment, we analyzed media collected at different time points by immunoblotting. We found that Genetespib did not alter the secretion of GFP1-10 (Supplementary Figure 3A) and α-Syn (Supplementary Figure 3B) from either control or USP19-overexpressing cells. The inhibition of Hsp90 was confirmed by the observation that polyubiquitinated proteins accumulated more dramatically in cells treated with Genetespib and a proteasome inhibitor (MG132) than with MG132 alone (Supplementary Figure 3C), as reported previously^24^. Collectively, these results demonstrate that Hsp90 is not involved in MAPS.

### USP19 interacts with HSC70

**Fig. 3:**
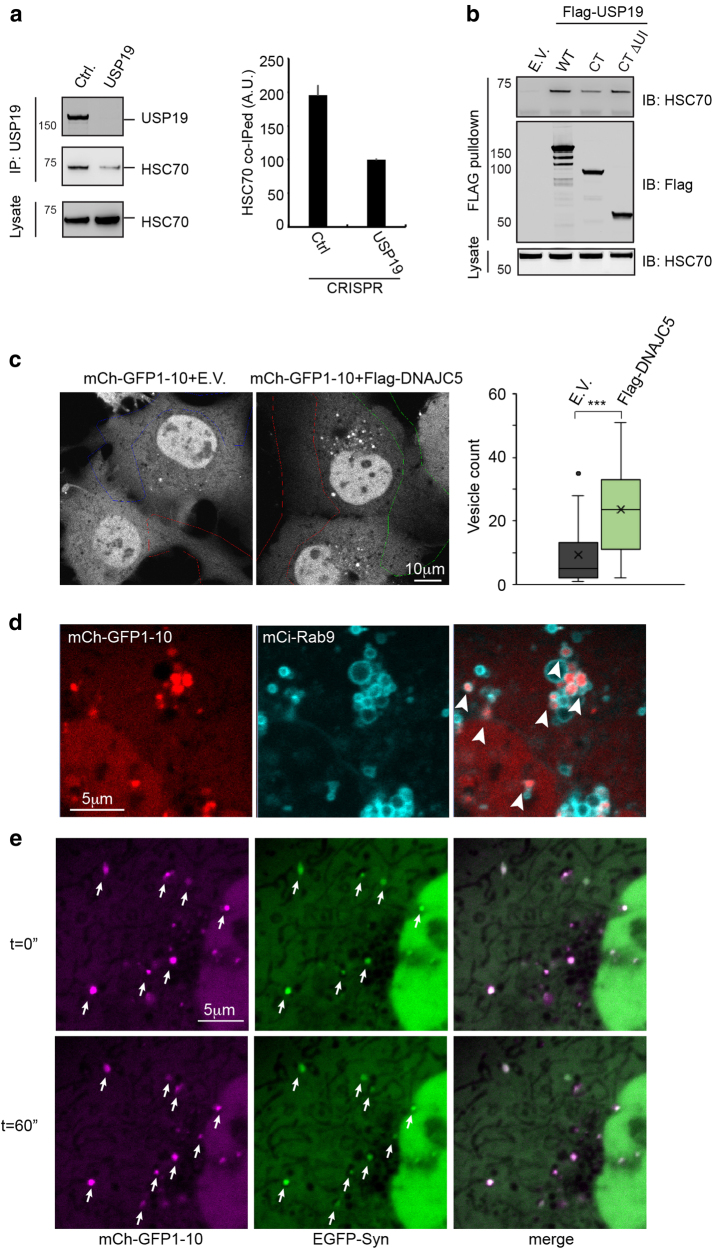
USP19 functions together with HSC70 and DNAJC5 in MAPS. **a** Endogenous USP19 interacts with HSC70. USP19 immunoprecipitated (IP) from lysates of control (Ctrl.) or USP19 null CRISPR cells was analyzed by immunoblotting. The graph shows the quantification of two independent experiments. **b** USP19 interacts with HSC70 via the USP domain. **c** DNAJC5 overexpression increases the entry of mCherry-GFP1-10 (mCh-GFP1-10) into vesicles in a perinuclear region. COS7 cells transfected with the indicated plasmids together with mCitrine-Rab9 (mCi-Rab9) for 16 h were subject to 5 rounds of photobleaching in the indicated regions. The graph shows the number of GFP1-10 vesicles in the absence and presence of DNAJC5 (mean ± s.e.m., ***p < 0.005, cell number *n* = 20 for control and 21 for USP19-expressing cells). **d** DNAJC5 induces the entry of GFP1-10 into vesicles that contain Rab9. Cells co-transfected with mCh-GFP1-10, mCi-Rab9, and Flag-DNAJC5 were photobleached and imaged. **e** Colocalization of mCh-GFP1-10 with EGFP-α-Syn. Dual color photobleaching was performed using cells expressing both mCh-GFP1-10 and EGFP-α-Syn together with Flag-DNAJC5. Cells were then imaged by time lapse fluorescence confocal microscopy

In addition to Hsp90, HSC70 was co-precipitated with Flag-tagged USP19 overexpressed in HEK293T cells^5^. However, whether HSC70 interacts specifically with endogenous USP19 in untransfected cells was unclear. To test this, we performed co-IP using USP19 antibodies. Because HSC70 is the most abundant cytosolic protein, non-specific interaction with antibodies was observed^5^, which needs to be carefully controlled. An unrelated IgG control was inappropriate because different IgG species had different levels of background binding. We therefore used a CRISPR cell line lacking endogenous USP19 as a negative control. Cell extracts were incubated with affinity-purified USP19 antibodies immobilized on protein A beads. Bound materials were analyzed by immunoblotting, which demonstrated that immunoprecipitation of USP19 consistently co-precipitated more HSC70 from WT cells than from USP19 deficient cells (Fig. 3a), suggesting an interaction between these proteins under endogenous conditions. Mapping experiments using the above-mentioned USP19 variants showed that the interaction was preserved with a C-terminal (CT) fragment that lacks UI (Fig. 3b). Coincidentally, this corresponds to the minimal USP19 fragment sufficient for MAPS stimulation.

Because HSC70 forms a stable complex with its co-chaperone DNAJC5, which was recently implicated in unconventional secretion of Tau and α-Syn^15^, we also tested whether USP19 could interact with DNAJC5. Co-IP showed that Flag-tagged USP19 co-precipitated with a small fraction of ectopically expressed DNAJC5 (Supplementary Figure 4A), but endogenous DNAJC5 was not found in complex with USP19 (Data not shown). We concluded that USP19 does not form a stable interaction with DNAJC5.

### DNAJC5 promotes protein secretion via the MAPS pathway

The observed interaction of USP19 with HSC70 prompted us to investigate whether DNAJC5–HSC70 uses the MAPS pathway to enhance Tau and α-Syn secretion. Because the function of HSC70 and DNAJC5 in UPS was only characterized using Tau, TDP43, and α-Syn as substrate, we first checked whether the previously established MAPS model substrate GFP1-10 could be secreted in a DNAJC5-dependent manner. We showed previously that a fraction of GFP1-10 was recruited to the ER surface by USP19 and subsequently enters into a population of Rab9 positive endosomes for secretion^3^. Likewise, when DNAJC5 was co-expressed with GFP1-10 in cells, it induced GFP1-10 secretion significantly (Supplementary Figure 4B, C), suggesting that DNAJC5 may also be a MAPS regulator.

If DNAJC5 stimulates protein secretion via MAPS, overexpression of DNAJC5 should result in more cargo-bearing endosomes similarly to USP19 overexpression. To test this possibility, we used a previously established photobleaching-based live cell imaging assay. To this end, COS7 cells transfected with mCherry-tagged GFP1-10 (mCh-GFP1-10) together with a control or DNAJC5-expressing plasmid were subject to repeated photobleaching to quench fluorescence signal in ~30% of the cytosolic area (Fig. 3c). Because mCh-GFP1-10 diffuses rapidly in the cytosol, this procedure diminished its signal throughout the cytoplasm, allowing visualization of mCh-GFP1-10 trapped within endosomes out of the bleaching area. As reported previously, in cells expressing mCh-GFP1-10 alone, we observed ~10 substrate-bearing endosomes per cell^3^, but co-expression of DNAJC5 led to more such structures (Fig. 3c). When these cells were co-transfected with mCitrine-Rab9 (mCi-Rab9) to label late endosomes, 169 out of 189 mCh-GFP1-10-containing endosomes in 11 cells analyzed were decorated with Rab9, revealing their identity as the MAPS compartment (Fig. 3d). In addition, when we performed dual color photobleaching using cells transfected with mCh-GFP1-10, EGFP-α-Syn and DNAJC5, we found that ~80% mCh-GFP1-10-containing endosomes also carried EGFP-α-Syn (Fig. 3e), suggesting that these substrates use the same vesicle carrier to exit cells. Collectively, these results strongly suggest that DNAJC5 and USP19 function in the same UPS pathway.

### Overexpression of DNAJC5 promotes MAPS independently of USP19

**Fig. 4:**
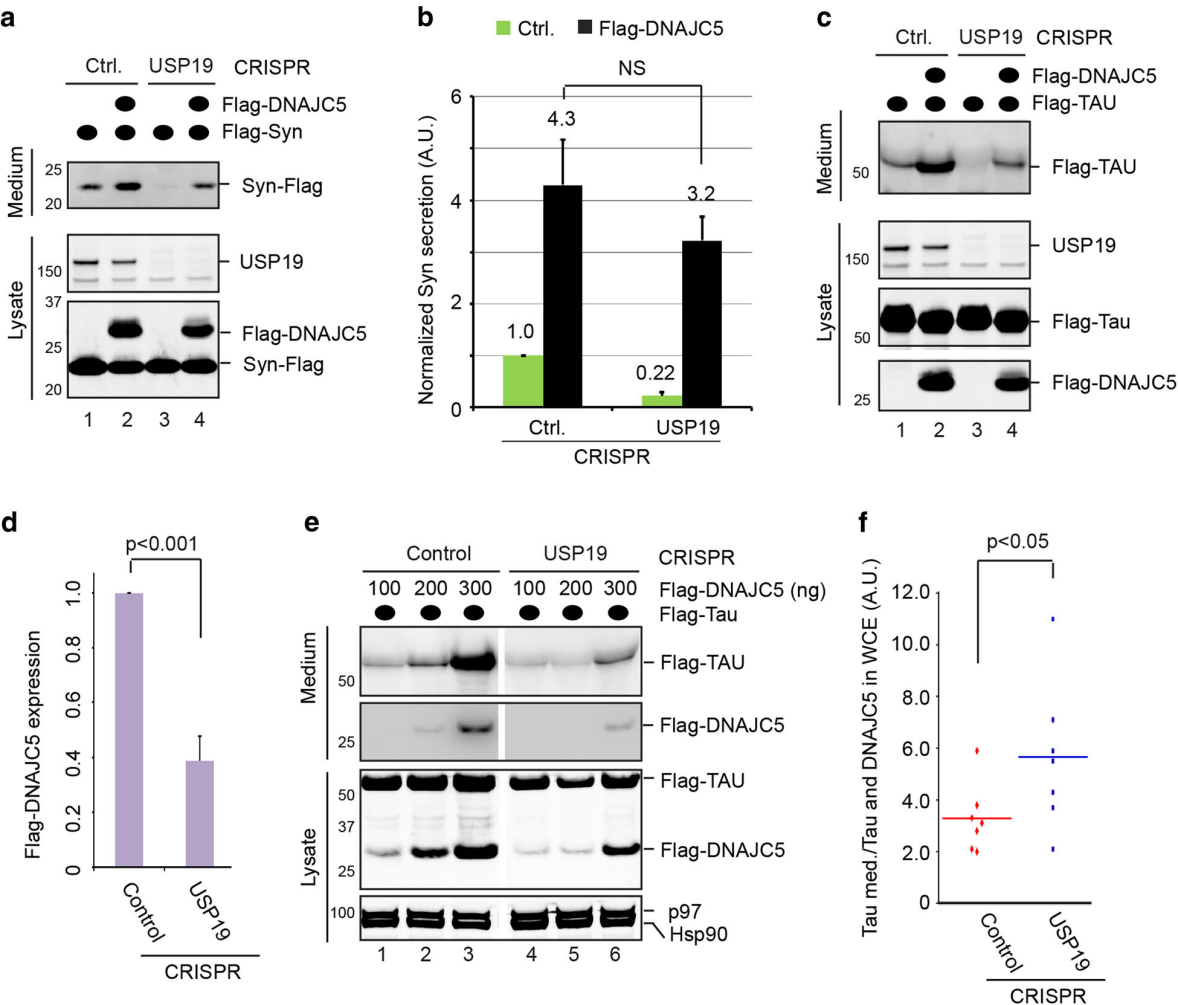
DNAJC5-stimulated MAPS is independent of USP19. **a** DNAJC5 induces α-Syn secretion in both WT control (Ctrl.) and USP19 null CRISPR 293T cells. **b** The graph shows the quantification of three independent experiments in (**a**) (mean ± s.e.m., *n* = 3). For DNAJC5-overexpressing cells, the level of secretion is further normalized by DNAJC5 expression in cell lysate using control CRISPR cells as the reference. **c** DNAJC5 induces Tau secretion in both WT and USP19 null cells. **d** Flag-DNAJC5 expression in WT and USP19 null cells as determined by quantitative immunoblotting (mean ± s.e.m., *n* = 6). **e** As in (**c**) except that different amount of DNAJC5 plasmid were used. **f** Knockout of USP19 does not reduce DNAJC5-induced Tau secretion. Shown is the relative amount of Tau in medium (med.) normalized first by Tau and then by DNAJC5 in whole cell extract (WCE) from seven transfections in three independent experiments

To elucidate the functional interplay between USP19 and DNAJC5-HSC70 in MAPS, we clarified the epistatic link between these factors. We first tested whether HSC70-DNAJC5 might function upstream of USP19. If so, depletion of USP19 should abolish DNAJC5-stimulated secretion. To this end, control or USP19 null CRISPR cells were transfected with α-Syn together with an empty vector or DNAJC5-expressing plasmid. Immunoblotting analyses showed that in control cells, DNAJC5 overexpression enhanced α-Syn secretion by ~4-fold (Fig. 4a, lane 2 vs. 1, Fig. 4b), consistent with a previous report^15^. In USP19 null cells, basal α-Syn secretion was reduced by ~80% compared to WT cells (lane 3 vs. 1), but DNAJC5 overexpression was still able to stimulate α-Syn secretion, albeit to a lower level than that in control cells (lane 4 vs. 2). Although similar results were obtained with Tau and GFP1-10 (Fig. 4c; Supplementary Figure 4B), we noticed that in USP19 CRISPR cells, the level of ectopically expressed DNAJC5 was only ~35% of that in control cells (Fig. 4d). When we normalized the secretion of α-Syn and GFP1-10 by DNAJC5 levels, we did not observe significant difference between control and USP19 null cells (Fig. 4b; Supplementary Figure 4C), suggesting that USP19 is not required for DNAJC5-stimulated secretion. To further substantiate this conclusion, we re-examined Tau secretion using control or USP19 null cells transfected with different amount of DNAJC5 plasmid. We compared Tau secretion from cells expressing similar levels of DNAJC5 (Fig. 4e, lane 6 vs. 2), which was further normalized by DNAJC5 expression. The results confirmed that DNAJC5-stimulated Tau secretion was not reduced by depletion of USP19 (Fig. 4f). Altogether, these results suggest that DNAJC5 is either downstream of USP19 or independent of it.

### USP19 acts upstream of HSC70-DNAJC5 in MAPS

**Fig. 5:**
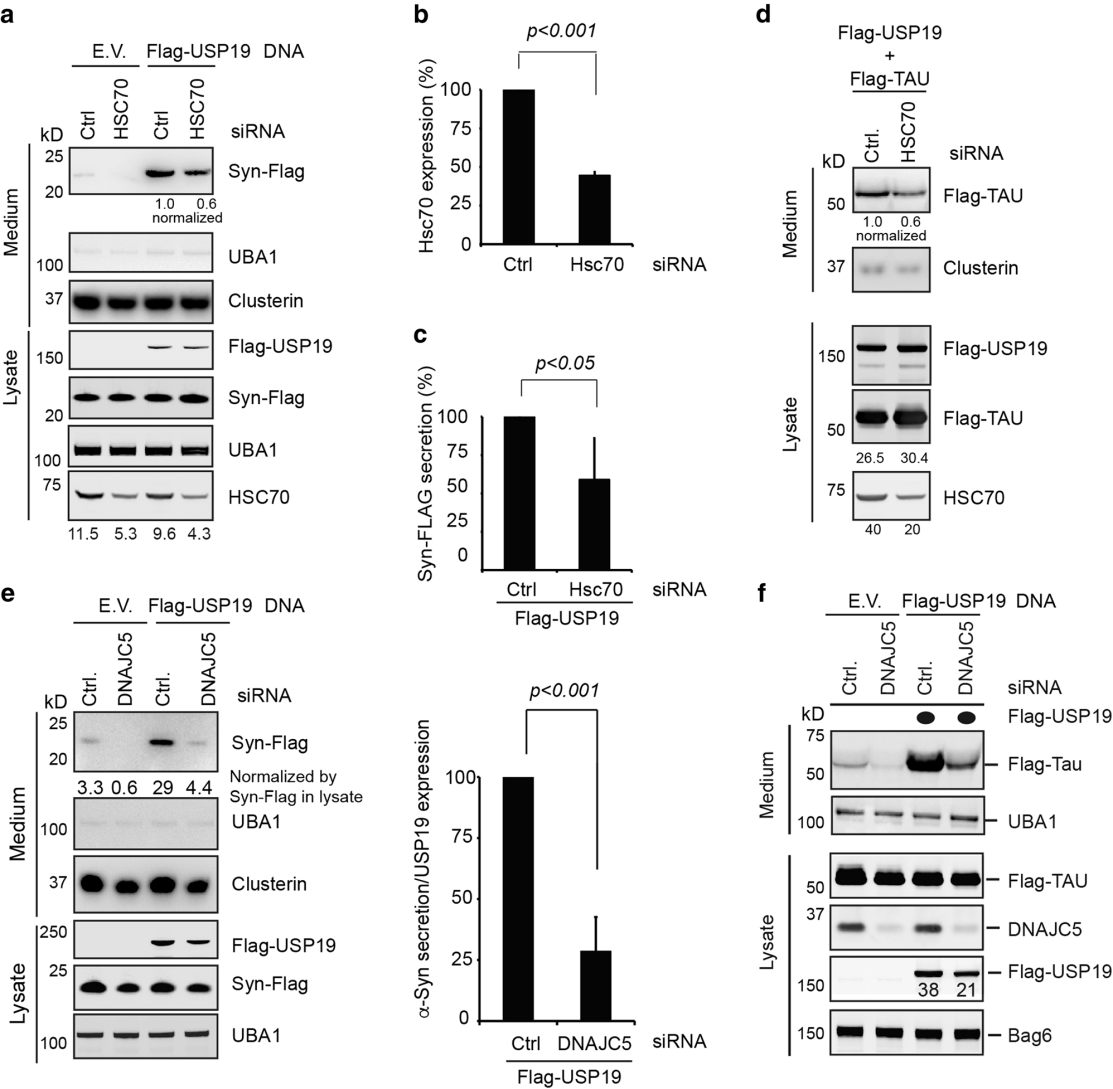
HSC70 and DNAJC5 function downstream of USP19 in MAPS. **a** Knockdown of HSC70 reduces USP19-induced secretion of α-Syn. **b** HSC70 knockdown efficiency in the secretion experiments as determined by quantitative immunoblotting analyses (mean ± s.e.m., *n* = 3). **c** Quantification of α-Syn secretion in control (Ctrl.) and HSC70 knockdown cells that expressed USP19. Shown is the level of α-Syn secretion normalized by α-Syn in lysate (mean ± s.e.m., *n* = 3). **d** HSC70 knockdown reduces Tau secretion from cells overexpressing USP19. **e** Knockdown of DNAJC5 reduces USP19-mediated secretion of α-Syn. As in (**a**), except that DNAJC5 was knocked down. The graph shows the relative levels of α-Syn secretion from control and DNAJC5 knockdown cells that also express USP19. α-Syn in media is normalized by α-Syn and USP19 in lysate (mean ± s.e.m., *n* = 3). **f** DNAJC5 knockdown reduces USP19-induced Tau secretion. As in (**e**), except that F-Tau was expressed instead of α-Syn

We next used a similar approach to test whether USP19 acts upstream of HSC70 and DNAJC5. Because HSC70 is an essential gene, we used siRNA to transiently silence its expression. Due to its abundance, we were only able to reduce the HSC70 protein level by ~55% in siRNA-transfected cells (Fig. 5a, b). Nevertheless, this was sufficient to reduce basal secretion of α-Syn (Fig. 5a, lane 2 vs. 1), as reported previously^15^. Knockdown of HSC70 also reduced the secretion of α-Syn by ~40% under USP19-overexpressing conditions (Fig. 5a, lanes 3, 4; Quantified in c). USP19-induced secretion of Tau and GFP1-10 was also similarly diminished by HSC70 knockdown (Fig. 5d; Supplementary Figure 5A). Under these conditions, USP19 was expressed at similar levels between control and HSC70 knockdown cells. We therefore concluded that efficient induction of MAPS by USP19 requires HSC70. Since depletion of USP19 reduced the interaction of GFP1-10 with HSC70 (Supplementary Figure 5B), USP19 seems to promote substrate binding by HSC70.

We next tested whether DNAJC5 is required for USP19-stimulated MAPS by knocking down DNAJC5 in USP19-overexpressing cells. Quantitative immunoblotting showed that depletion of DNAJC5 resulted in ~70% reduction in α-Syn and Tau secretion in USP19-overexpressing cells (Fig. 5e, f). Although DNAJC5 knockdown reduced the expression of USP19 by ~30%, this was unlikely to account for the difference in MAPS efficiency between control and DNAJC5 knockdown cells for the following reasons. First, titration experiments showed that the effect of USP19 on MAPS was saturable (Supplementary Figure 1A). Under our experimental conditions, 50% reduction in USP19 expression did not significantly reduced α-Syn secretion (Supplementary Figure 5C, lane 3 vs. 2). Second, when we titrated the USP19 plasmid to achieve similar levels of expression in control and DNAJC5 knockdown cells, α-Syn secretion was still significantly reduced by depletion of DNAJC5 (Supplementary Figure 5C, lane 6 vs. 2). Altogether, these results suggest that USP19 functions upstream of HSC70 and DNAJC5 in MAPS.

### DNAJC5 is localized to late endosomes to promote MAPS

To further understand the role of DNAJC5 in MAPS, we characterized its subcellular localization. The human genome contains three DNAJC5-related genes that are highly similar in sequence (also named CSP, CSPβ, and CSPγ). They all contains a string of cysteine residues (CS) known to be palmitylated, which target these proteins to membranes^25^. However, the precise localization of these proteins in cells remains controversial. While several studies suggested that CSP proteins are localized to synaptic vesicles or secretory granules^26^, one study reported CSPβ as a Golgi resident protein (Boal et al., 2007)^27^.

**Fig. 6:**
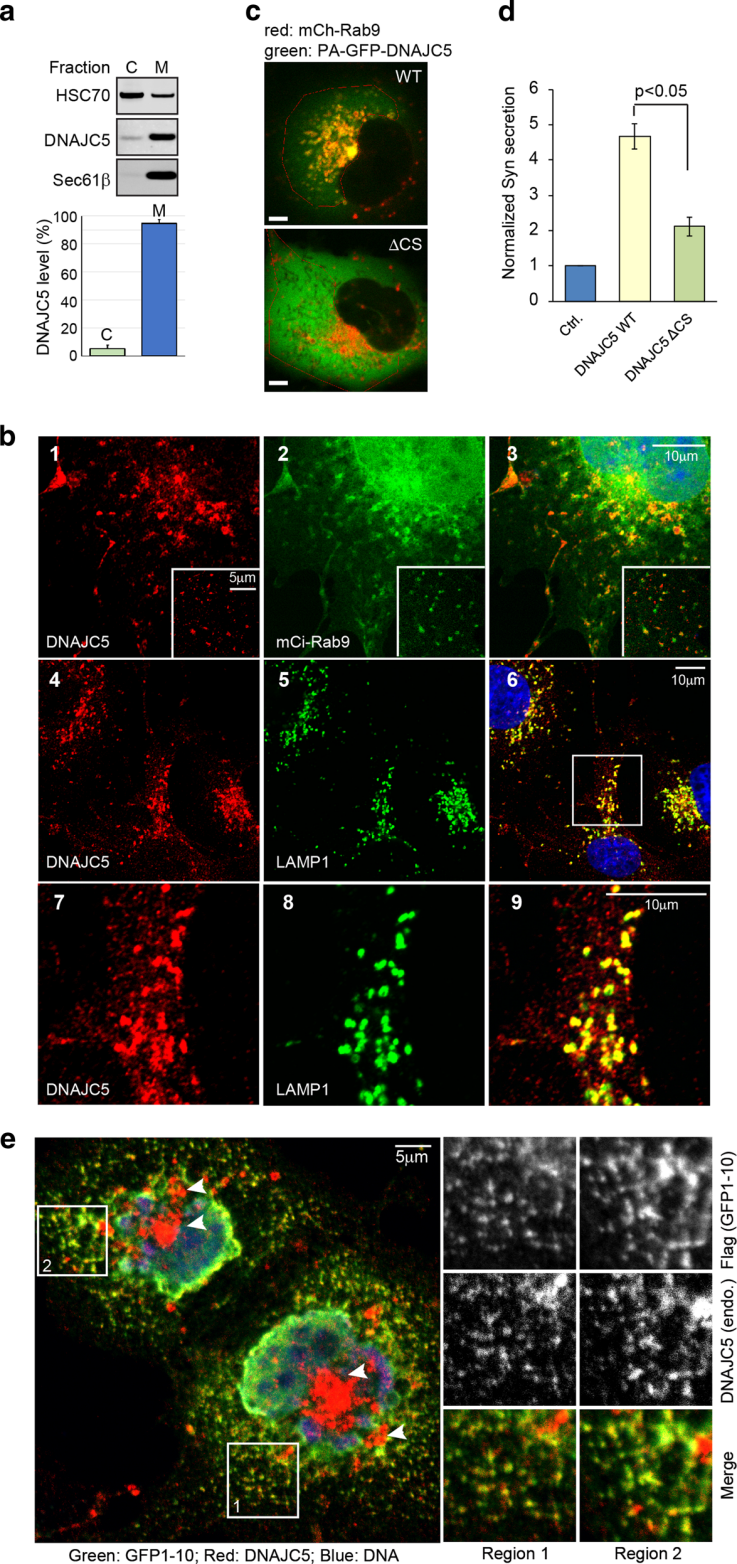
DNAJC5 is a late endosome-associated protein that promotes membrane association of a MAPS substrate. **a** Endogenous DNAJC5 is mostly associated with membranes. 293T cells were fractionated into a cytosol (C) and a membrane (M) fraction for analysis by quantitative immunoblotting (IB) (mean ± s.e.m., *n* = 3). **b** Endogenous DNAJC5 is localized to compartments positive for Rab9 or LAMP1. 1–3 COS7 cells transfected with mCitrine-Rab9 (mCi-Rab9) were stained with a DNAJC5 antibody in red. The insets show an example of DNAJC5 co-localization with Rab9. 4–6, COS7 cells were stained with DNAJC5 antibody in red and LAMP1 antibody in green. 7–9 is an enlarged view of the boxed area in panel 6. **c** Membrane association requires the cysteine string domain (CS) of DNAJC5. COS7 cells expressing mCherry-Rab9 (mCh-Rab9) together with photoactivable (pA) GFP-tagged wild type (WT) DNAJC5 or a mutant lacking the CS segment were imaged after activation of DNAJC5 fluorescence in the indicated areas. **d** The CS domain is required to stimulate MAPS. Secretion of α-Syn from 293T cells expressing the indicated proteins was quantified and normalized to the levels of DNAJC5 in lysates (mean ± s.e.m., *n* = 3). **e** Endogenous DNAJC5 is co-localized with a MAPS substrate. Cells expressing mCerulean-USP19 (mCe-USP19) and mCh-GFP1-10 were permeabilized, fixed and stained with DNAJC5 in red, Flag in green and DAPI in blue. Arrowheads indicate lysosome-localized DNAJC5, which is not colocalized with GFP1-10. The two indicated regions that contain many small DNAJC5-positive vesicles were enlarged and shown in the right panels

To resolve the controversy, we first performed cell fractionation to determine the localization of endogenous DNAJC5 in 293T cells. The result showed that ~90% of endogenous DNAJC5 was present in membrane fractions (Fig. 6a). We then performed immunostaining to determine the subcellular localization of endogenous DNAJC5 in COS7 cells using a DNAJC5-specific antibody (Supplementary Figure 6A). We found that endogenous DNAJC5 displays two types of membrane localization: it is present in both small vesicles throughout the cytoplasm and large punctae in a perinuclear region (Supplementary Figure 6B, panel 1). These membrane compartments do not contain the early endosome marker EEA1, and do not overlap with either the Golgi markers GalT and GM130 or the ER marker Calreticulin (Supplementary Figure 6B). Instead, most of them are decorated with the late endosome marker mCi-Rab9 (Fig. 6b, panels 1–3). Intriguingly, only large peri-nuclear punctae positive for DNAJC5 could be stained by an antibody against the lysosome-associated membrane protein 1 (LAMP1) (Fig. 6b, panels 4–9). These results suggest that DNAJC5 is localized to both late endosomes and lysosomes.

Since ectopically expressed DNAJC5 stimulates MAPS, if membrane association is important for DNAJC5’s function in MAPS, overexpressed DNAJC5 should not only be present at endosomes and lysosomes, but also recruits MAPS substrates to this compartment. Biochemical fractionation showed that a fraction of recombinant DNAJC5 also bound to membranes, but more was in the soluble cytosol fraction (Supplementary Figure 7A), suggesting that membrane binding is either saturable or regulated by a limiting factor. Nevertheless, this is sufficient to recruit a MAPS substrate to membranes (Supplementary Figure 7A). To see if membrane-associated recombinant DNAJC5 was localized to the correct compartment like the endogenous one, we generated DNAJC5 tagged with a photoactivatable GFP (PA-GFP-DNAJC5), which allowed us to label selectively membrane-associated DNAJC5 in live cells. Dual color confocal live cell microscopy showed that PA-GFP-DNAJC5 was indeed accumulated in vesicles positive for mCh-Rab9 (Supplemental Video 1; Fig. 6c). To determine whether endosome association was required for DNAJC5-mediated MAPS, we generated a mutant DNAJC5 that lacks the CS domain. As anticipated, this variant was mostly localized to the cytosol (Fig. 6c). Compared to WT DNAJC5, this mutant showed significantly reduced activity in promoting α-Syn secretion (Fig. 6d). Thus, late endosome localization is critical for the function of DNAJC5 in MAPS.

To further determine the functional significance of late endosome-associated DNAJC5 in MAPS, we determined the localization of GFP1–10, a MAPS substrate in digitonin-treated cells that expressed mCerulean-USP19 (mCe-USP19). Digitonin treatment permeabilized the plasma membrane and thus removed soluble cytosolic proteins, allowing visualization of the remaining membrane-bound GFP1-10. Because USP19 promotes the entry GFP1-10 into the MAPS pathway^3^, if this is mediated by a DNAJC5-containing membrane compartment, GFP1-10 should be co-localized with endogenous DNAJC5 at this compartment. Interestingly, membrane-associated Flag-GFP1-10 showed extensive co-localization with endogenous DNAJC5 in small endosomal vesicles (Fig. 6e, right panels), but not at the peri-nuclear DNAJC5-positive compartment that corresponds to lysosomes (Fig. 6e). Similar results were obtained when we imaged mCh-GFP1-10 after photobleaching in cells expressing PA-GFP-DNAJC5 in live cells (Supplementary video 2). Because no significant mCh-GFP1-10 signal was seen in untransfected cells, we excluded the possibility that vesicle-associated GFP1-10 had resulted from uptake of released GFP1-10 (Data not shown). These findings are consistent with our photobleaching studies, which showed the entry of several MAPS substrates into the Rab9-positive compartment^3^. Altogether, we conclude that DNAJC5 promotes MAPS via late endosomes.

### Co-secretion of DNAJC5 with misfolded polypeptides

**Fig. 7:**
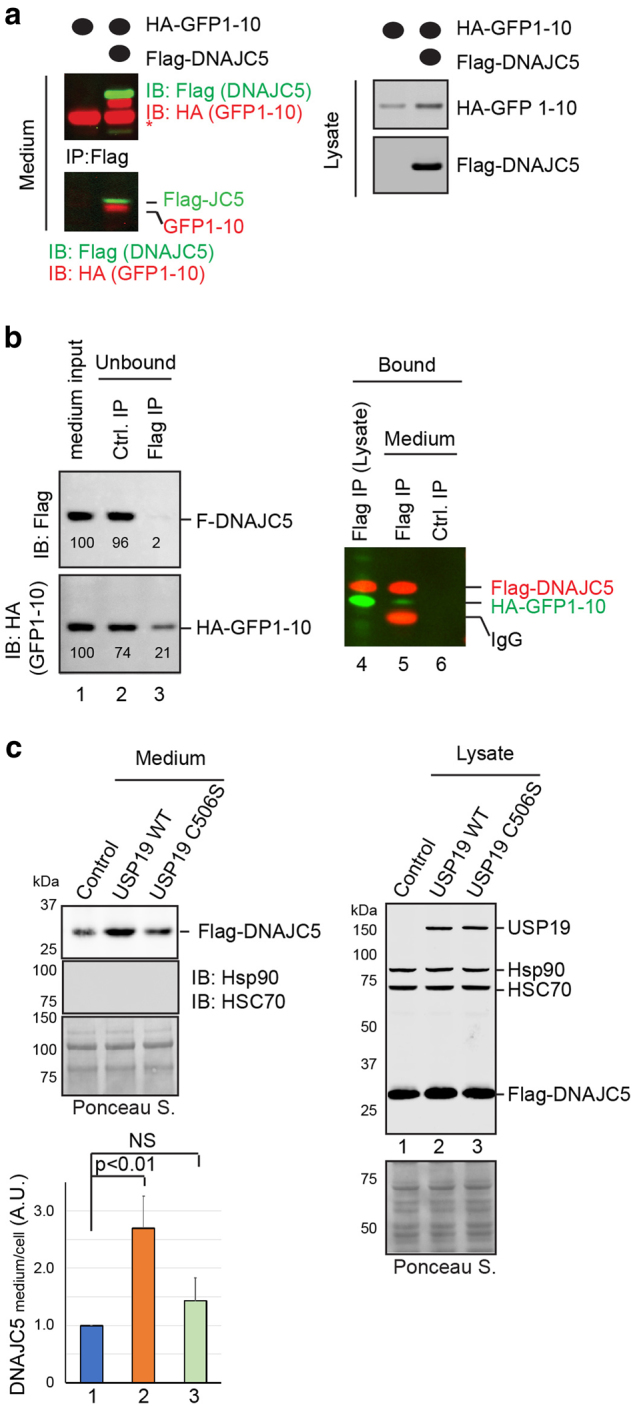
DNAJC5 is co-released with MAPS substrates. **a** Interaction of DNAJC5 with secreted GFP1-10 in conditioned media. Conditioned media from transfected 293T cells was either directly analyzed by immunoblotting (IB), or first subject to IP by a Flag antibody before IB. Asterisk indicates the IgG bands. **b** Most GFP1-10 molecules secreted are in complex with DNAJC5. Conditioned media were either directly analyzed by IB (input), or first separated into a DNAJC5-positive and a DNAJC5 free fraction by IP with Flag antibody before IB. **c** USP19 enhances DNAJC5 secretion. Secretion of FALG-DNAJC5 from cells co-transfected with the indicated plasmids was analyzed. Where indicated, cell lysates were also analyzed by immunoblotting. The graph shows normalized DNAJC5 secretion (mean ± s.e.m., *n* = 3)

While characterizing the function of DNAJC5 in MAPS, we noticed that a fraction of overexpressed DNAJC5 was also secreted (Fig. 4e). Since in cells DNAJC5 co-localizes and interacts with MAPS substrate (Fig. 6e; Supplementary Figure 7A), we reasoned that DNAJC5 might chaperone MAPS substrates to the cell exterior. To test this hypothesis, we first performed co-IP using conditioned medium collected from cells transfected with HA-GFP1-10 and Flag-DNAJC5. As a negative control, Flag-DNAJC5 was omitted. IP with Flag antibodies showed that without DNAJC5, no GFP1-10 was precipitated, but when medium from cells co-expressing GFP1-10 and DNAJC5 was used, GFP1-10 could be readily detected together with DNAJC5 in precipitated samples (Fig. 7a). Reciprocal immunoprecipitation using GFP antibody to pull down GFP1-10 further confirmed that at least a fraction of secreted GFP1-10 stayed in a complex with DNAJC5 (Supplementary Figure 7B). A similar experiment also detected secreted α-Syn in complex with DNAJC5 (Supplementary Figure 7C).

To better gauge the level of association between secreted MAPS substrates and DNAJC5, we analyzed both the immunoprecipitated samples as well as the unbound fractions using cells expressing Flag-DNAJC5 and HA-GFP1-10. Compared to control IP using Sepherose beads, Flag-beads efficiently depleted Flag-DNAJC5 from the medium (>90%), which resulted in co-depletion of GFP1-10 by more than 60% (Fig. 7b). Moreover, we found that WT USP19 but not USP19 C506S also enhanced DNAJC5 secretion (Fig. 7c), consistent with the idea that when USP19-induced MAPS, DNAJC5 accompanied substrates to the cell exterior. Altogether, we conclude that in MAPS misfolded proteins are escorted to the cell exterior by cellular chaperones like DNAJC5.

### Intercellular transmission of a MAPS substrate

**Fig. 8:**
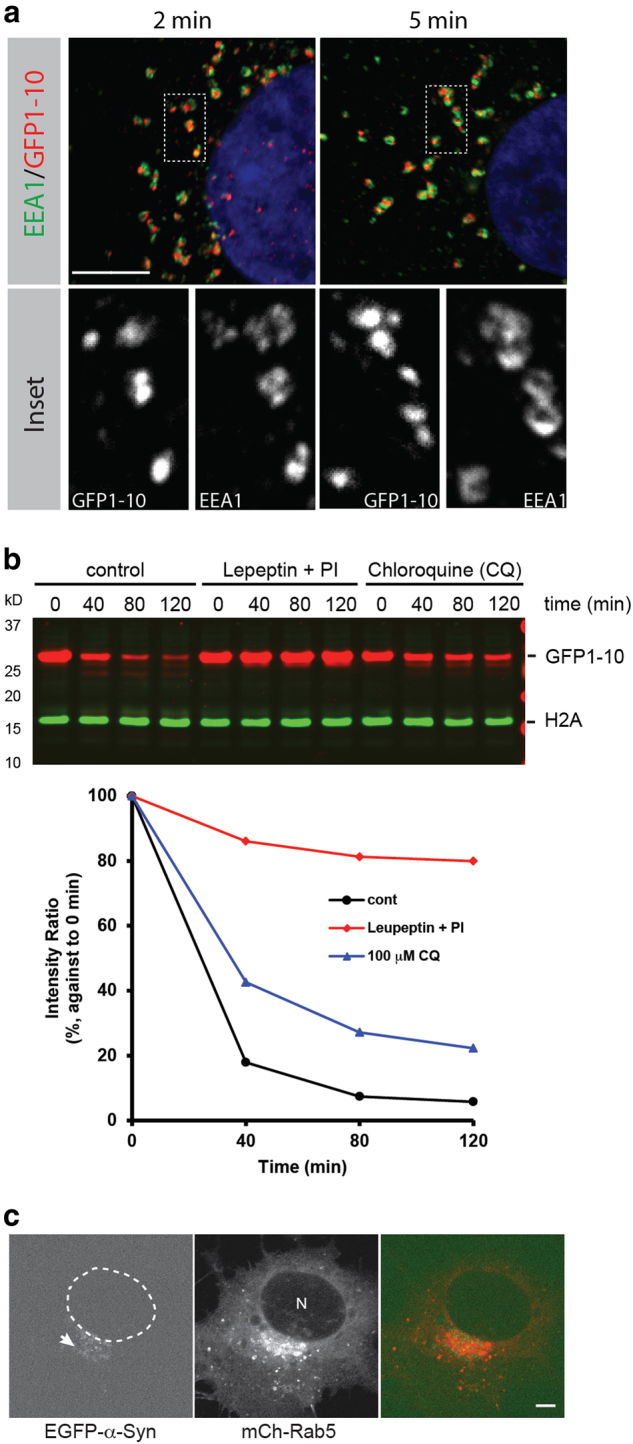
Uptake and degradation of a misfolded protein by endocytosis. **a** GFP1-10 is taken up into EEA1 positive early endosomes. Recombinant GFP1-10 was incubated with COS7 cells at 37 °C for the indicated time periods. Cells were stained with an EEA1 antibody (green) and GFP antibody (red). **b** Degradation of recombinant GFP1-10 by lysosomes. 293T cells were incubated with GFP1-10 and then washed. Cells were incubated in fresh medium in the presence of the indicated inhibitors. At the indicated time points, a fraction of the cells were lysed and analyzed by IB. The gel shown is a representative of 3 independent experiments. The graph shows the quantification of the experiment. PI, protease inhibitors. **c** Uptake of secreted α-synuclein by COS7 cells. EGFP-α-Syn-expressing cells were transfected with Flag-DNAJC5. Conditioned medium between 34 and 48 h post-transfection was harvested and concentrated by 5-fold. COS7 cells transfected with mCh-Rab5 were then incubated with conditioned medium for 2 h before live imaging by a confocal microscopy. The dashed line indicates the nucleus (N). The arrow indicates peri-nuclear vesicles where internalized α-Syn is localized. Scale bars, 5 µm

Upon secretion, misfolded proteins may ultimately aggregate in the extracellular space if not removed in a timely manner. We postulated that eukaryotic cells might also possess a mechanism that internalizes secreted MAPS substrates for degradation by the lysosome. To test this hypothesis, we first used GFP1-10 as a model substrate because it could be readily purified in a soluble form. We incubated recombinant GFP1-10 purified from *E. coli* with COS7 cells. Shortly after incubation, GFP1-10 was observed in small vesicles that were also positively labeled by antibodies against the early endosome marker EEA1 (Fig. 8a). After a longer chase, these vesicles appeared to become juxtaposed to lysosomes labeled by LAMP1 (Supplementary Figure 8A), suggesting that internalized GFP1-10 is being targeted to the lysosomes. Immunoblotting analysis of cell extracts taken at different time points after removal of free unbound GFP1-10 confirmed that GFP1-10 entering cells was short-lived (Fig. 8b). The degradation of GFP1-10 could be blocked by a lysosomal protease inhibitor cocktail or attenuated by chloroquine, which interferes with lysosomal degradation by affecting the pH of the lysosomes (Fig. 8b). By contrast, the proteasome inhibitor MG132 had no effect (Supplementary Figure 8B). These observations suggest that mammalian cells can effectively remove misfolded proteins from the extracellular space via endocytosis.

To see if misfolded proteins secreted through MAPS could also be taken up by cells, we treated COS7 cells with conditioned media harvested from 293 T cells overexpressing EGFP-α-Synuclein and DNAJC5. Live cell imaging indeed detected green fluorescence signals in cells after incubation, which were partially co-localized with the early endosome marker Rab5 (Fig. 8c; Supplemental video 3). These results confirm that misfolded proteins secreted via MAPS can undergo cell-to-cell transmission.

## Discussion

### Role of the USP19 and DNAJC5 in MAPS

In this study, we demonstrate that the MAPS pathway can be used to dispose an array of neurotoxic proteins. The secretion efficiency of individual proteins varies, but for every substrate tested, USP19 overexpression increases the secretion. How cells select misfolded proteins for secretion through the MAPS pathway is not entirely clear. Since misfolded proteins usually have exposed hydrophobic residues, these hydrophobic elements probably serve as the initial signal to engage membrane-anchored USP19 given the previously established chaperone activity for USP19^3^. Structural studies have revealed that α-Syn contains a *N*-terminal helical hairpin and an extended unstructured C-terminal region^28^. The latter likely constitutes the site for the initial recognition by USP19. The structure of Tau is unknown, but given that the fusion of GFP to its *N*-terminus abolishes Tau secretion, the secretion signal might be spatially in proximity to the *N*-terminus of Tau, which might be masked when the bulky GFP tag is appended. The secretion signal for MAPS probably to some extent overlaps with ‘degrons’ that target many of these abnormal proteins for proteasomal degradation. Consistent with this view, several MAPS substrates are known to undergo ubiquitination^3, 4, 29, 30^. Deubiquitination by USP19 may be sufficient to alter their fate from degradation to secretion. For substrates that are insufficiently ubiquitinated, it is possible that the deubiquitinating activity of USP19 may become dispensable for secretion.

**Fig. 9:**
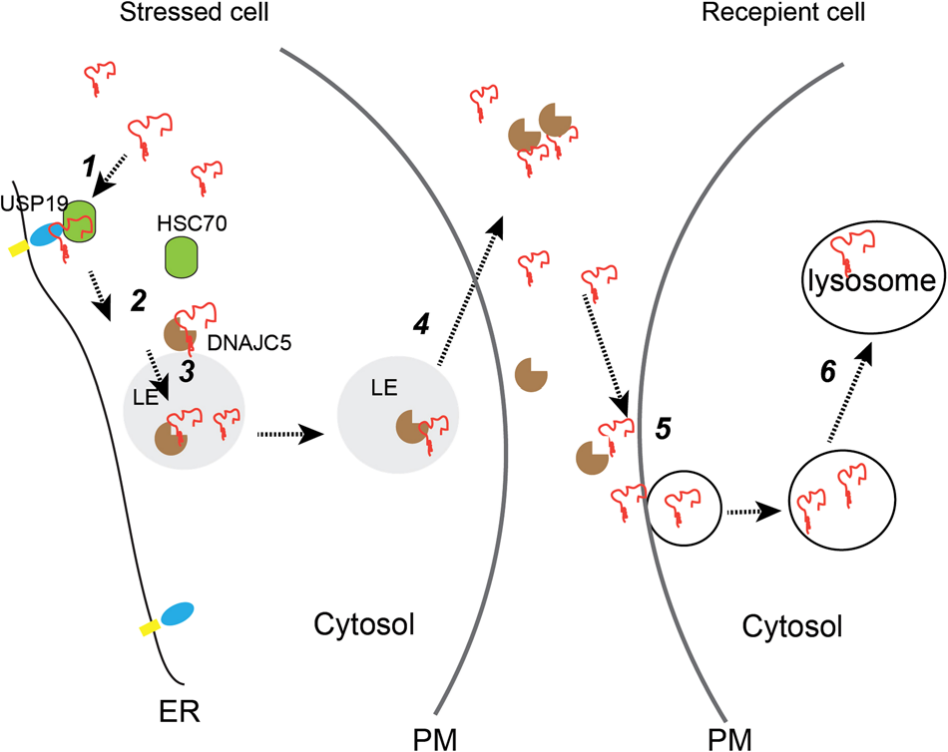
A coordinated PQC mechanism allows cells to share a proteotoxic stress burden. A model of protein quality control via intercellular transfer of misfolded proteins. PM, plasma membrane; ER, endoplasmic reticulum; LE, late endosome. Misfolded cytosolic proteins can be secreted by the following steps. In step 1, misfolded proteins (in red) are recruited to the ER surface by binding to a receptor (e.g., USP19) that has a chaperone activity. In step 2, misfolded proteins are transferred to DNAJC5, a HSC70 co-chaperone that is associated with the membrane of LEs and lysosomes, which are in close contact with the ER. In step 3, the complex of DNAJC5 and misfolded proteins are moved into the lumen of late endosomes probably via a protein translocation channel. In step 4, LEs contact and fuse with the PMs to release misfolded proteins. Once secreted, misfolded proteins can be taken up by another cell (Step 5) and degraded in the lysosomes (Step 6)

Because DNAJC5 and HSC70 function downstream of USP19, aberrant polypeptides may be transferred from USP19 to DNAJC5 prior to secretion (Fig. 9). This is reminiscent of a recently reported UPS path that exports an ER-retained misfolded protein to the cell surface. In this process, the chaperones HSC70 and its co-chaperone DNAJC14 are required^31^, but it is unclear whether USP19 is involved. We show that DNAJC5 is predominantly localized to late endosomes and lysosomes. Thus, MAPS is probably initiated when misfolded proteins are recruited by USP19 to the ER surface before being transferred to DNAJC5 for secretion (Fig. 9). Since MAPS substrates are not expected to enter the ER lumen, the transport of these substrates from the ER to late endosome-associated DNAJC5 should not be dependent on vesicular transport. Consistent with this model, secretion of MAPS substrates is not sensitive to brefeldin A, a drug that blocks the formation of ER-derived COPII vesicles^3^. By contrast, conditions that disturb endocytic trafficking have been shown to enhance α-Synuclein secretion^32^, in line with the notion that MAPS substrates flux through a late endocytic compartment prior to secretion.

### MAPS and other unconventional protein secretion pathways

It is long known that many eukaryotic proteins lacking signal sequence can nevertheless exit the cell, apparently through an UPS path^8, 10, 33, 34^. In the last decade, several UPS cargos have been characterized. Among them, with the exception of a few, the remaining ones seem to use vesicle intermediates to reach the cell exterior, but the vesicles involved were thought to be different, resulting in what appears to be different forms of UPS^9, 34^. For instance, in autophagy-mediated secretion of IL1β and other cargos, substrates enter the lumen of a LC3-positive compartment via an unclear translocation mechanism^35–37^. For other cargos, some were proposed to enter late endosomes via multivesicular bodies and subsequently released into the extracellular space in small vesicles termed exosomes^38^.

The MAPS pathway is clearly distinct from exosome-mediated secretion because cargos secreted by MAPS are not bound to vesicles^3^. MAPS also differs from autophagy-mediated secretion in several aspects. A major one is the chaperone requirement. Autophagy-mediated secretion of IL1β requires Hsp90^35^, whereas MAPS is dependent on the HSC70-DNAJC5 complex. Moreover, autophagy-dependent secretion is initiated from a peri-nuclear region where the ER and Golgi are concentrated, and the Golgi resident protein GRASP was proposed to form a specialized UPS compartment for this process^39, 40^. By contrast, GRASP is not required for MAPS (Lee et al., 2016). Despite these differences, MAPS and autophagy-mediated UPS do share some common features. First, cargos are thought to be translocated into the lumen of a membrane compartment, autophagasome for IL1β and late endosomes for MAPS cargos^3, 35^. As autophagasomes constantly fuse with late endosomes during maturation, the protein translocation machinery for the two processes might be identical. Second, although autophagasomes and late endosomes are both destined to the lysosomes to turn over carried contents, cargos of these two UPS pathways do not seem to succumb to this fate, at least not completely. Instead, cargoes are released into the cell exterior probably due to interaction and fusion of these secretory vesicles with the plasma membrane. In this regard, it is tempting to speculate that eukaryotic cells may contain a unique population of autophagasome/endosome dedicated for UPS. Further studies are required to elucidate the functional relationships between these UPS pathways.

### Role of MAPS in PQC and neurodegeneration

Cells defective in MAPS are hyper-sensitive to proteasome inhibitor treatment, indicating MAPS as a vital PQC mechanism that enhances cell’s overall capacity to cope with proteotoxic stress^3^. We now show that aberrant proteins secreted by MAPS can be taken up by non-stressed cells and degraded by the lysosomes. This mechanism presumably allows cells to avoid accumulating misfolded proteins in the extracellular space despite the constitutive action of MAPS. Although it is unclear whether stressed cells can also take up and degrade misfolded proteins from the extracellular space, our study suggests a novel PQC paradigm in which over-stressed cells “share” misfolded proteins with healthy neighbor cells, soliciting help from the latter to clear aberrant polypeptides (Fig. 9). This process might be particularly critical for the viability of neurons because mutations in DNAJC5 are known to cause ceroid lipofuscinosis,^41, 26^ a neurodegenerative condition characterized by accumulation of auto-fluorescent “lysosome-like materials”. These lysosomal materials might be remnants of MAPS vesicles, accumulated due to defects in secretion. Misfolded proteins entering recipient cells might escape degradation and exit the endocytic system. When this occurs, they may serve as seeding templates to induce protein misfolding in recipient cells, resulting in propagation of misfolded polypeptides. Further studies are warranted to determine whether the MAPS pathway contributes to the intercellular transmission of misfolded polypeptides under aging or disease conditions, which if proven to be true, might offer a completely new perspective to develop unprecedented therapeutic schemes for diseases such as Parkinson’s disease and Alzheimer’s disease.

## Materials and methods

### Cell lines, siRNAs, and plasmids

HEK293 and COS7 cells were purchased from ATCC. USP19 CRISPR null and control cells were described previously^3^. The cells were maintained in DMEM medium (Corning cellgro) containing 10% fetal bovine serum and penicillin–streptomycin. Cells recovered from liquid nitrogen freezing were maintained for at least 2 weeks before being used in the secretion experiments. The sources of mammalian expression constructs, chemicals, proteins, and antibodies are listed in Supplementary Table 1. To generate pA-GFP-DNAJC5, full-length rat DNAJC5 (identical amino acid to the human counterpart) was cloned into pPA-GFP C1 (Addgene #11910) vector using BglII and SalI restriction sites. To construct plasmids expressing different USP19 truncation variants, USP19-encoding DNA fragments listed below were PCR-amplified from the pRK5-USP19-WT-Flag plasmid containing USP19 isoform 1 (NP_006668). The fragments were inserted into the pRK5 vector digested by SalI and NotI.

1. USP19 CT ∆TM (490-1287)
2. USP19 CT ∆UI (490-680 and 1058-1318)
3. USP19 CT ∆TM ∆UI (490-680 and 1058-1287)

To make plasmid expressing Flag-TAU, the TAU ORF sequence was amplified and ligated into the vector pcDNA5-FRT/TO, which was engineered to carry a Flag tag encoding sequence at the *N*-terminus. The TAU ORF was amplified from pRK5-EGFP-Tau (Addgene #46904) with the following primers:

5′-ACGGGGTACCGCTGAGCCCCGCCAGGAGTTC-3′

5′-ATCCGCTCGAGTCACAAACCCTGCTTGGCCAGGGA-3′.

To make plasmid expressing recombinant His-tagged GFP1-10 from E. coli, GFP1-10-encoding DNA was PCR-amplified from the pCMV–GFP1–10 plasmid using the following primers:

5′-ACGCGTCGACCAATGGTTTCGAAAGGCGAGGAG-3′;

5′-ATAAGAAGGCGGCCGCTTATTTCTCGTTTGGGTCTTTG-3′.

The PCR fragment was ligated into the pET28a vector (Novagene) between the SalI and NotI sites. The pRK-HA-GFP1-10 was constructed by ligating the GFP1-10 fragment into the SalI and NotI sites of a pRK vector that engineered with an HA tag between the HindIII and SalI sites.

siRNAs were purchased from Invitrogen. The targeting sequences for HSC70 and DNAJC5 are:

siRNA-HSC70: 5′- GAAGCUUGCCUUGAAAUAU-3′

siRNA-DNAJC5: 5′-GCUUACGGCUUAGACAAAA-3′

Cells stably expressing EGFP-α-Synuclein were generated by several rounds of fluorescence activated cell sorting following transfection with an EGFP- α-Synuclein-expressing plasmid.

Transfection was performed with TransIT-293 (Mirus) for HEK293T cells, and with Lipofectamine2000 (Invitrogen) for COS7 cells. Lipofectamine RNAimax was used according to the manufacturer’s protocol in all gene silencing experiments.

### Immunoblotting, protein level measurements and statistical analyses

Immunoblottings were performed using the standard protocols. To quantify proteins secreted into the media, 15 μl conditioned medium of each condition was directly analyzed by immunoblotting using HRP-conjugated secondary antibodies. Immunoblotting signal was detected by the enhanced chemiluminescence method (ECL) and recorded by a Fuji LAS-4000 imager. The intensity of the detected protein bands was quantified by ImageGauge v3.0. Protein secretion efficiency was determined by normalizing the level of secreted proteins by the amount of expressed proteins in cell lysates determined by immunoblotting with fluorescence labeled secondary antibodies. For immunoblottings using fluorescent-labeled secondary antibodies, blots were scanned by a LI-COR Odyssey scanner, and the intensity of protein bands was determined by the Odyssey software.

### Immunoprecipitation under native conditions

To detect the interaction of USP19 with HSC70 or DNAJC5 in cells, HEK293T cells (~ 5 × 10^5^) were seeded and grown in a 6-well plate for 24 h, and then transfected with 1 μg plasmid expressing the proteins indicated in the figure legends. Cells were collected 24 h post-transfection and lysed in 350 μl buffer LCHAPS containing 1% CHAPS, 30 mM Tris/HCl pH 7.4, 150 mM potassium acetate, 4 mM magnesium acetate, 1 mM DTT, and a protease inhibitor cocktail. Cleared cell lysates were incubated with Flag M2 beads to precipitate Flag-tagged proteins. Immunoprecipitated protein complexes were washed two times with the buffer WCHAPS (0.1% CHAPS, 30 mM Tris/HCl pH 7.4, 150 mM potassium acetate, 4 mM magnesium acetate), and then eluted with the Laemmli buffer for immunoblotting.

### Protein secretion experiments

To measure the secretion of misfolded substrates, cells (2.5 × 10^5^) seeded in a poly-D-lysine -coated 12 well plate were grown for 24 h, and then transfected with 250 ng plasmids expressing the indicated MAPS substrates together with 250 ng plasmids expressing MAPS regulators as indicated in the figure legends. At 24 h post-transfection, we replaced the medium with 1.5 mL fresh DMEM medium. Cells were grown for another 16 h before the conditioned medium was collected. The medium was subjected to sequential centrifugation, first at 1000 × *g* for 5 min to remove contaminated cells, and then at 10,000 × g for 30 min to remove cell debris. Cells were lysed in 200 μl buffer LNP. A fraction of the cell lysates and medium was then analyzed directly by SDS–PAGE and immunoblotting. Unless specified in figure legends, protein secretion is normalized by the expressed protein in cell lysates. To knock down DNAJC5 and HSC70, cells (0.4 × 10^6^) were seeded in 6-well plate on day 0. On Day 1, cells were transfected with siRNA (60 pmol) and incubated for another 24 h. On day 2, MAPS substrates were transfected and cells were maintained for 30 h. Cells were then incubated with fresh medium for another 16 h before the medium was collected for analysis by immunoblotting.

### Biochemical fractionation experiments

HEK293T cells (1 × 10^7^) were collected and washed with ice-cold PBS. Cells were then treated with 800 μl buffer LHP (10 mM Tris-HCl pH 7.4, 10 mM potassium chloride, 2 mM magnesium chloride, 1 mM DTT) containing a protease inhibitor cocktail on ice for 10 min before being homogenized by a dounce homogenizer. Sucrose was added to 250 mM to prevent damage of subcellular organelles and membrane vesicles. Homogenized cells were subjected to centrifugation at ×1000 g for 5 min to remove unbroken cells and nuclei. The supernatant fractions were further centrifuged at ×100,000 g for 30 min to sediment total microsome membrane vesicles. The membranes were washed with the PB buffer containing 250 mM sucrose before SDS–PAGE analysis or protease treatment analysis.

### Recombinant protein purification

To express His_6_-GFP1-10, the pET28a-His_6_-GFP1-10 construct was transformed in *E. coli* BL21(DE3) cells. The bacteria were grown in LB medium supplemented with 50 µg/mL Kanamycin at 37 °C. Expression of His_6_-GFP1-10 was induced by 1 mM IPTG for 4 h. The bacteria were harvested, lysed, and the recombinant protein was purified using Ni-NTA resin (Thermo Scientific) according to the manufacturer’s instructions. The eluted protein was further purified by size-exclusion chromatography using Superdex 200. Protein was flash-frozen in liquid nitrogen and stored at −80 °C.

### Protein uptake and degradation assay

For the protein uptake assay, COS7 cells seeded on coverslips were incubated with 25 µg/mL recombinant GFP1-10 for 2 or 5 min at 37 °C, immediately followed by washing in PBS and fixation with 2% paraformaldehyde. The fixed cells were then permeabilized and immunostained for GFP1-10 and EEA1 using anti-GFP (mouse, 1:100, Sigma, B2) and anti-EEA1 antibodies (rabbit, 1:50, Cell Signaling), respectively. The cells were further stained with goat anti-mouse IgG-Alexa568 and goat anti-rabbit IgG-Alexa488. Immunofluorescence images were acquired on an LSM 780 confocal microscope.

For the detection of lysosomal localization of GFP1-10, COS7 cells were pretreated with 50 mM ammonium chloride for 30 min at 37 °C to inhibit lysosomal degradation. After washing, cells were incubated with 25 µg/mL GFP1-10 for 60 min, followed by washing and fixation. The cells were then permeabilized and stained for GFP1-10 and LAMP1 with specific antibodies.

For the degradation assay, 4 × 10^6^ HEK293T cells were pre-treated with 125 µM leupeptin (Sigma L5793) together with a lysosomal protease inhibitor cocktail (Sigma P8340, 1:100 dilution) or 100 µM chloroquine (CQ) for 1 h at 37 °C. Cells were then washed and incubated with 20 µg/mL GFP1-10 in PBS for 30 min at 4 °C. After washing with ice-cold PBS, the cells were resuspended in 450 µL DMEM medium and 100 µL cells were divided into each well of a 96-well plate and incubated at 37 °C to allow internalization of cell-bound GFP1-10. At different time points, cells (100 µL) in a well were directly mixed with 200 µL PBS and 100 µL 4 × Laemmli sample buffer, and heated at 95 °C for 10 min. Cell extracts were then resolved by SDS–PAGE and immunoblotted with an anti-GFP antibody.

### Cell permeabilization and immunostaining

Cells-expressing mCh-GFP1–10 either alone or together with USP19 were washed with ice-cold PBS and then treated with the PB buffer containing 0.055% digitonin and 1 mM DTT plus a protease inhibitor cocktail for 30 s. Cells were washed with PBS extensively and then fixed with 4% paraformaldehyde. Fixed cells were washed and stained with the antibodies as indicated in the figure legends in PBS containing 5% fetal bovine serum and 0.2% saponin. Cells were imaged on an LSM 780 confocal microscope (Carl Zeiss Microscopy). The objective lens used was the Zeiss Plan-Apo 63×/1.4 Oil DIC.

To quantify cells with membrane-associated GFP1–10 (Supplementary Fig. 5b), COS7 cells were transfected with either GFP1–10 alone or GFP1–10 together with Flag–USP19 for 24 h. After permeabilization, cells were fixed, stained by GFP and Flag antibodies, and then imaged by a Zeiss Axiovert fluorescence microscope using a 63× oil immersion Plan-Apochromat objective (NA 1.4).

### Live confocal microscopy

To characterize the ER-associated vesicles that contain MAPS cargos, COS7 cells were seeded at 1.5 × 10^5^ per well in a 35 mm µ-dish coated with fibronectin (ibidi GmbH, Germany). A total of 1 μg plasmid (700 ng MAPS cargos, 200 ng USP19 variants, 100 ng Rabs) was transfected into cells using Lipofectamine2000 following the manufacturer’s instructions.

Photobleaching and live confocal microscopy were performed 24 h post-transfection. For live-cell imaging, cells were incubated with phenol red-free MEM medium at 37 °C in a live-cell incubation chamber. Cells were illuminated with a 568 nm laser at maximum intensity for 6 rounds. Photobleached area is ~1/4 or 1/3 of the total cytoplasmic region, usually on the side where few endosome vesicles are present. Live-cell imaging was performed using an LSM 780 confocal microscope. The pin hole was set so the section thickness is about 1 μm, allowing continuous focus on cell peripheral regions where the cytoplasm is thin. To inhibit lysosomal degradation, cells were pre-treated with leupeptin 125 μM (Sigma L5793) together with a lysosomal protease inhibitor cocktail (Sigma P8340, 1:100 dilution) for 30 min before imaging. Images were acquired using Zen (Zeiss) and processed using Photoshop (Adobe). Supplemental videos were generated using ImageJ.

### Statistics and reproducibility

All gels shown are representatives of at least two independent replicates. The *n* values in the graphs indicate the number of independent experiments. Error bars show mean ± s.e.m., and *p* values were calculated using paired Student’s *t* test.

## Electronic supplementary material

Supplementary Information(PDF 1523 kb)

Supplemental video 1

Supplemental video 2

Supplemental video 3
