# Supplementary material for: DNAJC5 facilitates USP19-dependent unconventional secretion of misfolded cytosolic proteins
Source: Cell Discov. 2018 Mar 6;4:11. doi: 10.1038/s41421-018-0012-7 (PMC5838229; doi:10.1038/s41421-018-0012-7)
Supplement: Supplementary file 1 — Supplementary Information(PDF 1523 kb) [file 41421_2018_12_MOESM1_ESM.pdf]

Supplementary Table 1

REAGENT	SOURCE	IDENTIFIER
**RECOMBINANT DNA**		
pRK5-Flag-USP19 WT		[1]
pRK5-Flag-USP19 CT		[1]
pRK5-Flag-USP19 CT ΔTM	This study	
pRK5-Flag-USP19 CT ΔUI ΔTM	This study	
pRK5-Flag-USP19 CT ΔUI	This study	
pRK5-Flag-GFP1-10		[1]
pCMV-Flag-Tau	This study	
pcDNA3-DNAJC5-Flag		[2]
pET28-His-GFP1-10	This study	
pRK-HA-GFP1-10	This study	
GFP Htt[Q25]	Ron Kopito's lab	[3]
GFP Htt[Q103]	Ron Kopito's lab	[3]
pRK-Atx3-Q84		[4]
pCMV6-α-Syn-DDK (α-Syn-F)	Origene	
pCMV6-Ubl4A-DDK (Ubl4A-F)	Origene	
mCherry-C1-GFP1-10		[1]
mCitrine-Rab9		[1]
EGFP-α-Syn		[1]
pA-GFP-DNAJC5	This study	
**siRNA**		
HSC70	ThermoFicher	S6986
DNAJC5	ThermoFicher	S37239
USP19		[1]
**CHEMICAL**		
MG132	Millipore	474790
Genetespib	Selleckchem	STA-9090
**ANTIBODY**		
USP19		[1]
HSC70	Santa Cruz	SC-7298
HSP90	Santa Cruz	SC-13119
DNAJC5	SynapticSystems	154003
GFP	Santa Cruz	SC-9996
Flag	Sigma	F-1804
HA	Sigma	H-3663

UBE1	Sigma	E-3152
β-COP	ThermoFicher	MA3-067
EEA1	BD Transduction	610457
EEA1	Cell signaling	2411S
p97	Fitzgerald	10R-P104A
LAMP1	Santa Cruz	SC-18821
GM130	Abcam	Ab169276
Clusterin	Santa Cruz	SC-5289
Mouse(AlexaFluor®488conjugated)	ThermoFicher	A11029
Mouse(AlexaFluor®568conjugated)	ThermoFicher	A11031
Mouse(AlexaFluor®680conjugated)	ThermoFicher	A21058
Mouse(HRP-conjugated)	Sigma	A4416
Rabbit(AlexaFluor®488conjugated)	ThermoFicher	A11034
Rabbit(AlexaFluor®568conjugated)	ThermoFicher	A11011
Rabbit(IRDye800Conjugated)	Rockland	611-132-003
Rabbit(HRP-conjugated)	Sigma	A6154

Supplementary Figures

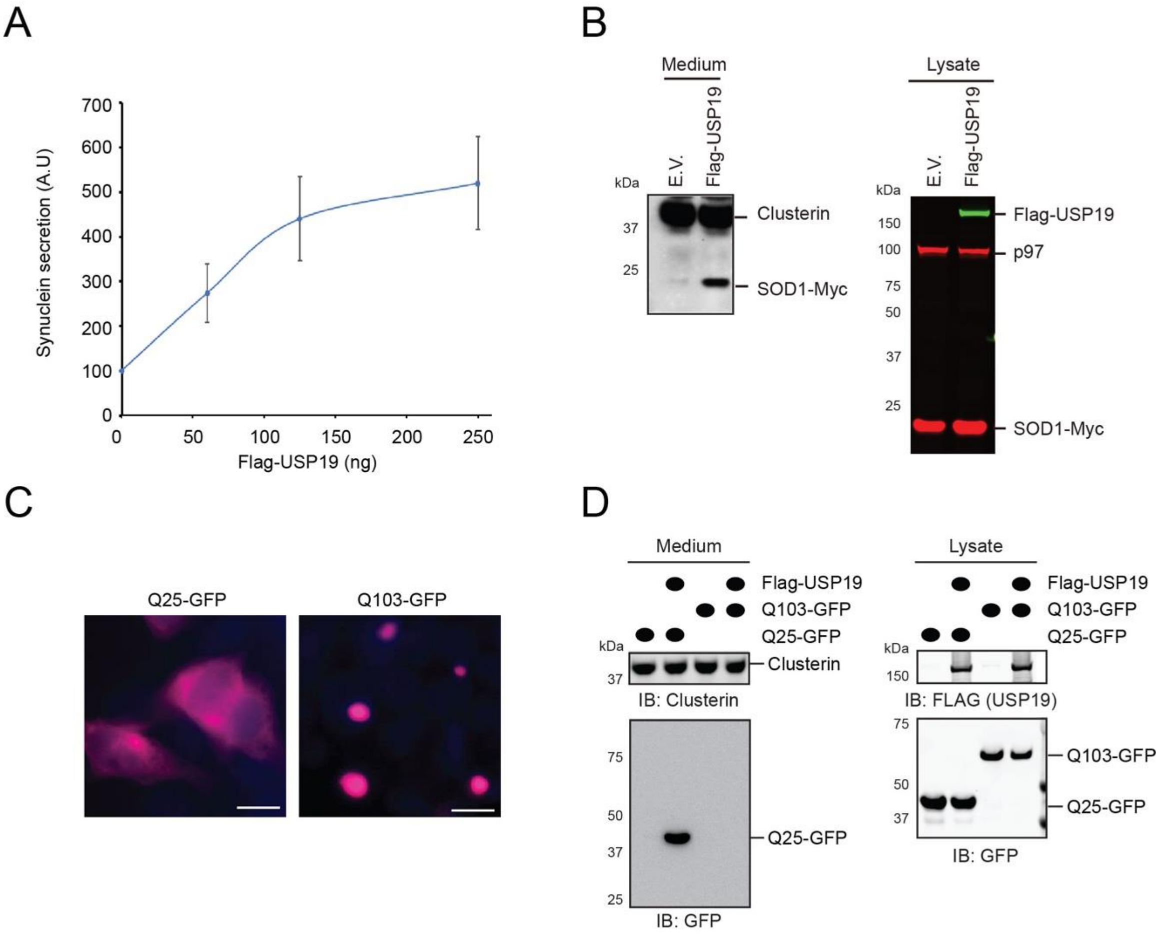

Figure S1 USP19 preferentially promotes secretion of soluble misfolded proteins

A, USP19 induces α-Syn secretion in a dose dependent manner in HEK293T cells. α-Syn secretion from cells transfected with α-Syn together with the indicated concentration of F-USP19 plasmid was normalized by α-Syn levels in cell lysate (mean ±s.e.m., n=3). **B**, USP19 overexpression promotes SOD1 secretion. E.V. empty vector. **C**, Q103-GFP forms large aggregates in HEK293 cells. Cells transfected with the indicated plasmids for 48 h were fixed and imaged. **D**, USP19 promotes the secretion of Q28-GFP, but no Q103-GFP was detected in the medium even when cells expressing USP19 were used.

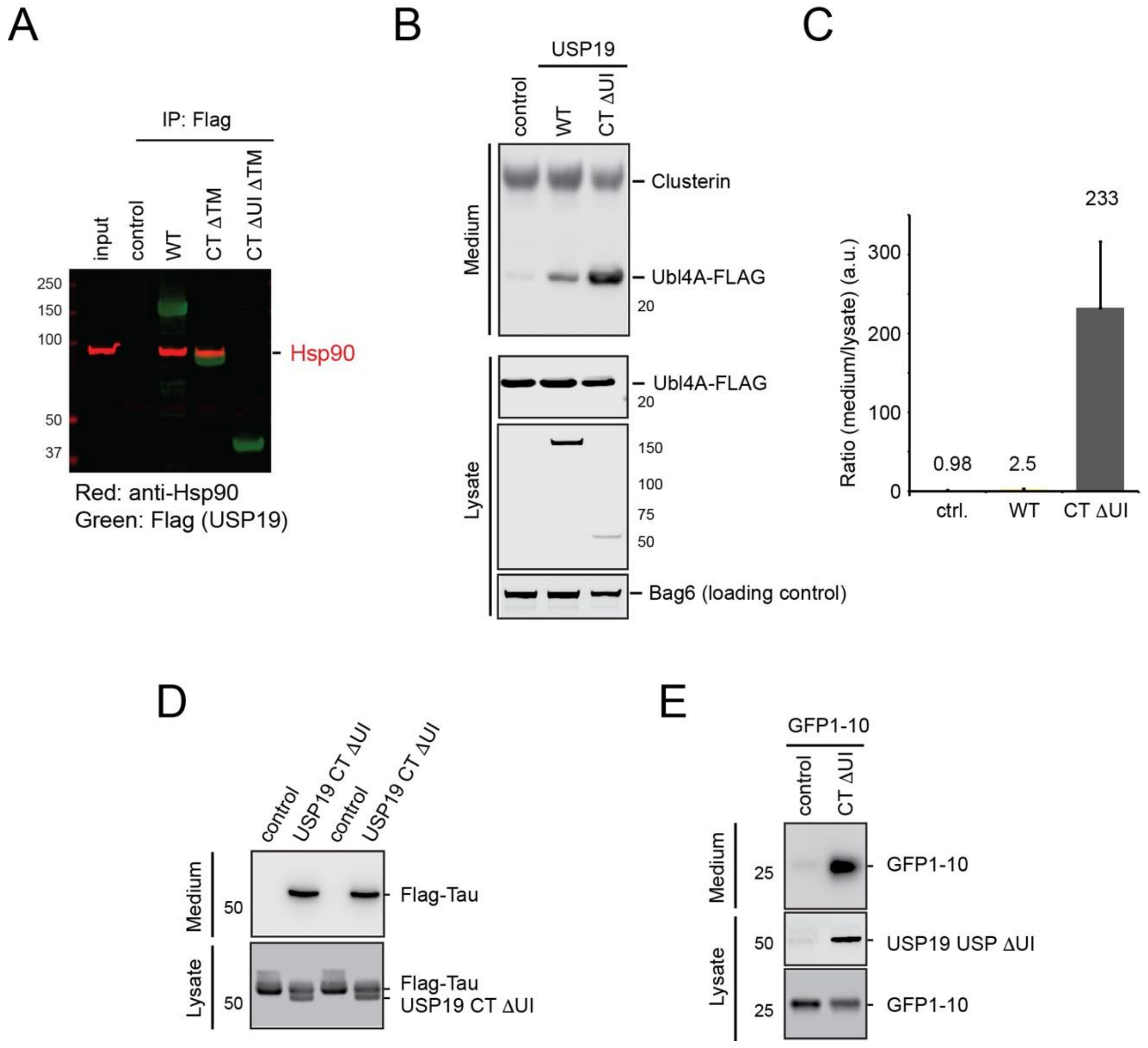

Figure S2 Hsp90 interaction is dispensable for USP19-stimulated secretion
A, Interaction of soluble USP19 variants with endogenous Hsp90. Cell lysates prepared from cell expressing the indicated USP19 variants were subject to IP followed by IB. As a negative control, cell transfected with an empty vector was used. **B**, **C**, A USP19 mutant lacking the UBL-containing insertion (UI) is a stronger MAPS stimulator. Secretion of unassembled Ubl4A-Flag from cells expressing the indicated USP19 variants was analyzed by IB. The graph in **C** shows the normalized secretion from 3 independent experiments (mean ±s.e.m., n=3). **D**, The USP19 CT ΔUI mutant also robustly stimulates Tau secretion. **E**, The USP19 CT ΔUI mutant promotes GFP1-10 secretion.

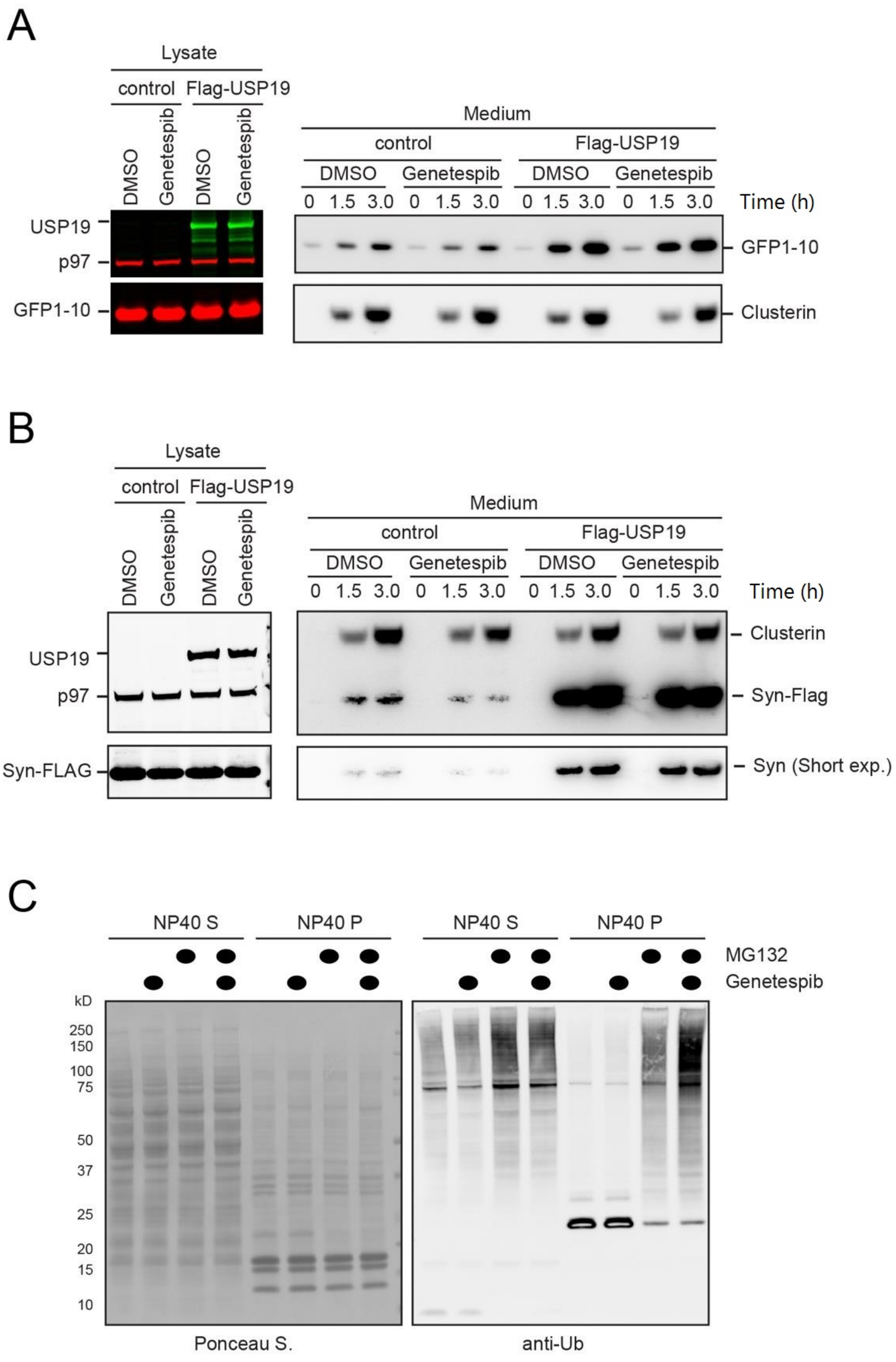

Figure S3 Hsp90 is not involved in MAPS

A, The Hsp90 inhibitor Genetespib does not block GFP1-10 secretion. 293T cells transfected with GFP1-10 together with USP19 or an empty control vector were treated with Genetespib. Conditioned medium harvested at the indicated time points was analyzed by IB. At the end of the chase, cell lysates were prepared

for IB analysis (left panels). **B**, Genetespib does not affect α-Syn secretion. As in **A**, except that cells expressing α-Syn-Flag were used. **C**, The efficacy of Genetespib was revealed by its ability to induce polyubiquitinated proteins in a detergent (NP40) insoluble fraction (S, soluble; P, insoluble pellet) in proteasome-inhibited cells.

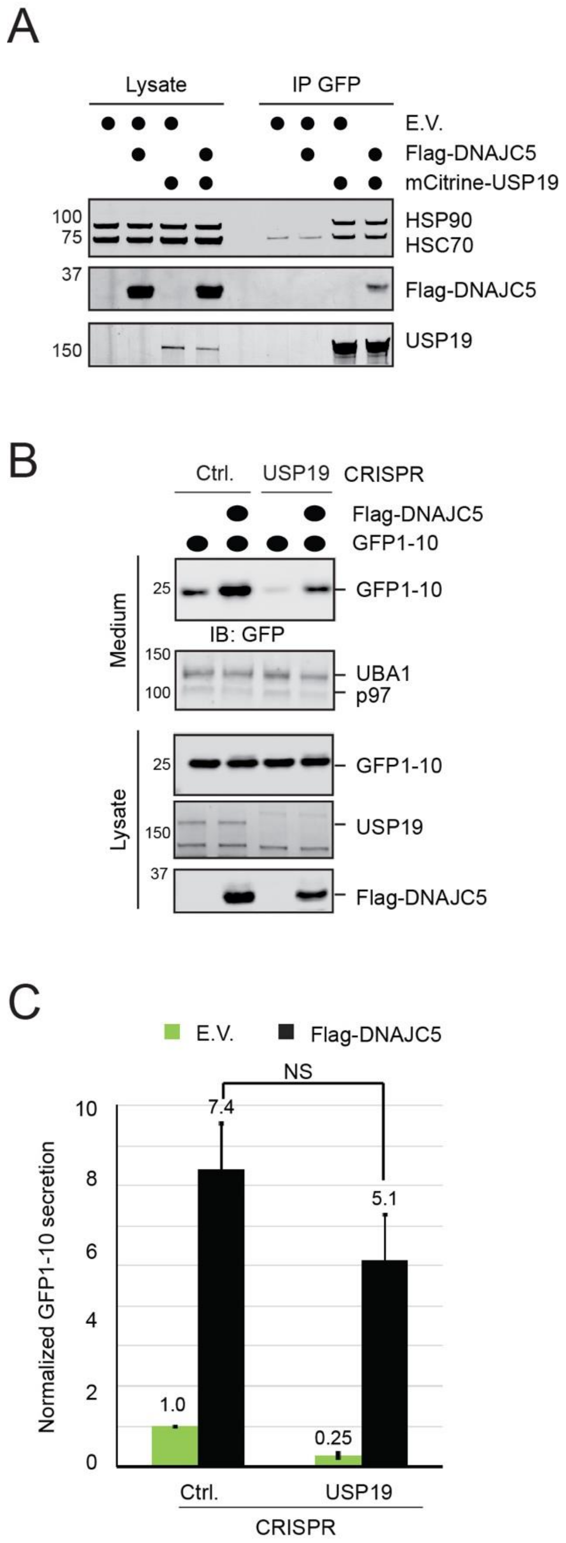
A
Lysate
IP GFP
E.V.
Flag-DNAJC5
mCitrine-USP19
100
75
HSP90
HSC70
37
Flag-DNAJC5
150
USP19
B
Ctrl.
USP19
CRISPR
Flag-DNAJC5
GFP1-10
Medium
25
GFP1-10
IB: GFP
150
100
UBA1
p97
Lysate
25
GFP1-10
150
USP19
37
Flag-DNAJC5
C
E.V.
Flag-DNAJC5
NS
Normalized GFP1-10 secretion
10
8
6
4
2
0
7.4
5.1
1.0
0.25
Ctrl.
USP19
CRISPR

Figure S4 DNAJC5-induced secretion does not require USP19

A, DNAJC5 does not promote the interaction of USP19 with HSC70. mCitrine-USP19 was immunoprecipitated from cells transfected with the indicated constructs and analyzed by IB. **B**, DNAJC5 induces GFP1-10 secretion in both control (Ctrl.) and USP19 null CRISPR cells. **C**, The graph shows the quantification of 3 independent experiments. For DNAJC-5 expressing cells, the relative secretion is normalized by DNAJC5 levels in cell lysate using the control cell as a reference (mean ±s.e.m., n=3, NS, not significant).

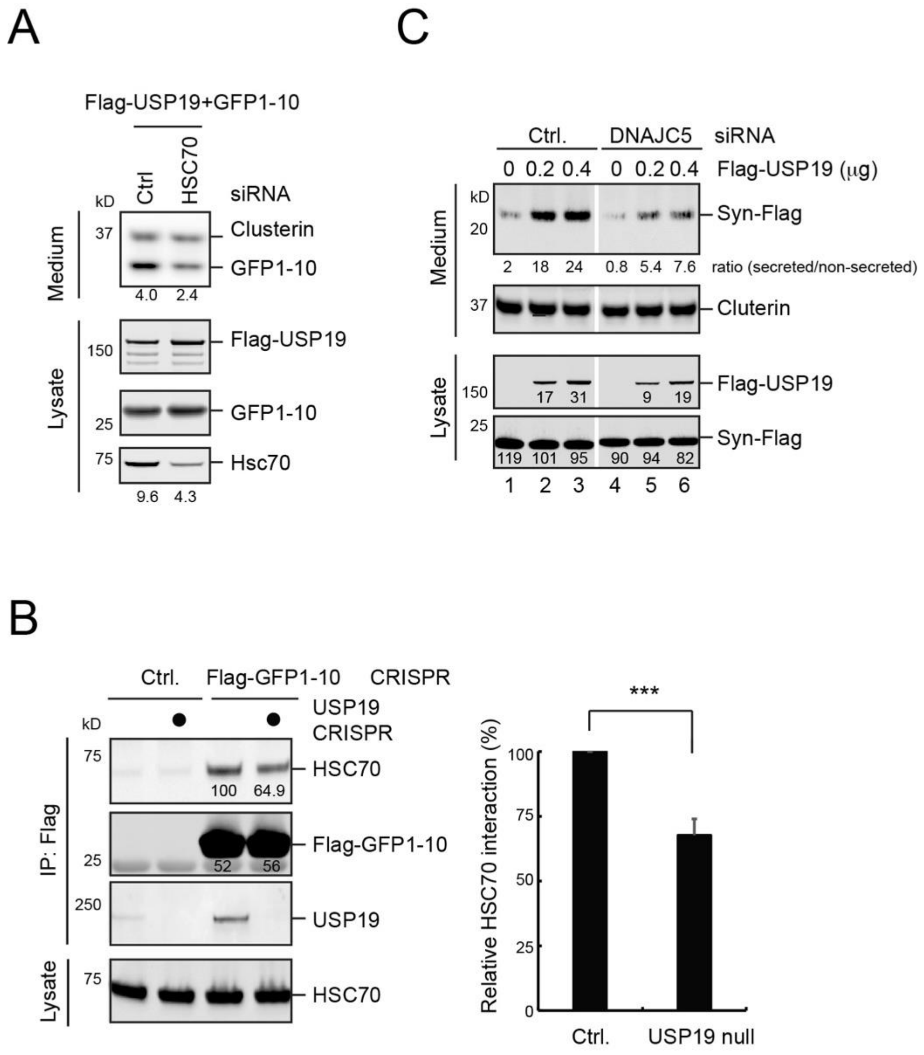

Figure S5 HSC70 and DNAJC5 function downstream of USP19

A, Knockdown of HSC70 reduces USP19-induced secretion of GFP1-10. **B**, Knockdown of DNAJC5 inhibits USP19-induced secretion of α-Syn. Where indicated, cells were transfected with different amount of USP19 DNA. Note that in cells expressing comparable level of USP19 (lane 6 vs. 2), knockdown of DNAJC5 reduced α-Syn secretion by ~60%. **C**, USP19 facilitates the interaction of GFP1-10 with HSC70. The graph shows the relative amount of substrate captured by HSC70 in control and USP19 null cell (mean ±s.e.m., ***, $p<0.001$, n=3).

A

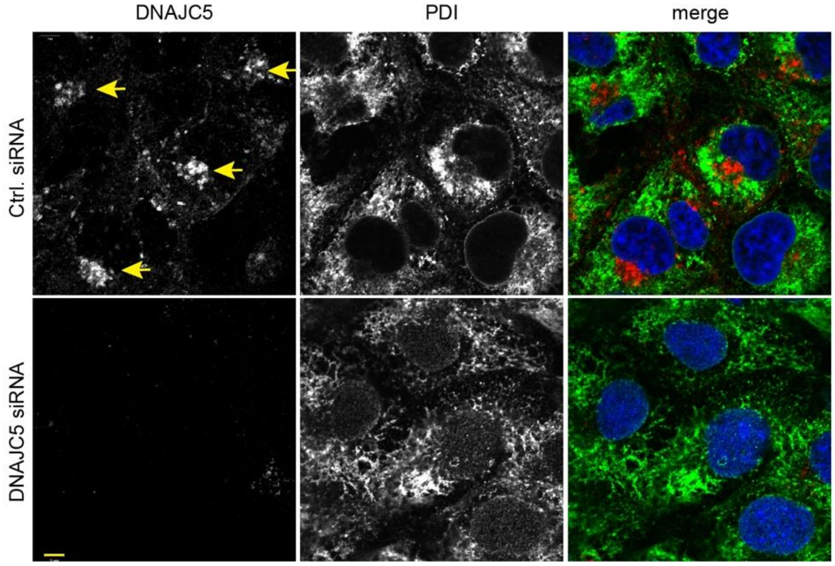
DNAJC5
PDI
merge
Ctrl. siRNA
DNAJC5 siRNA

B

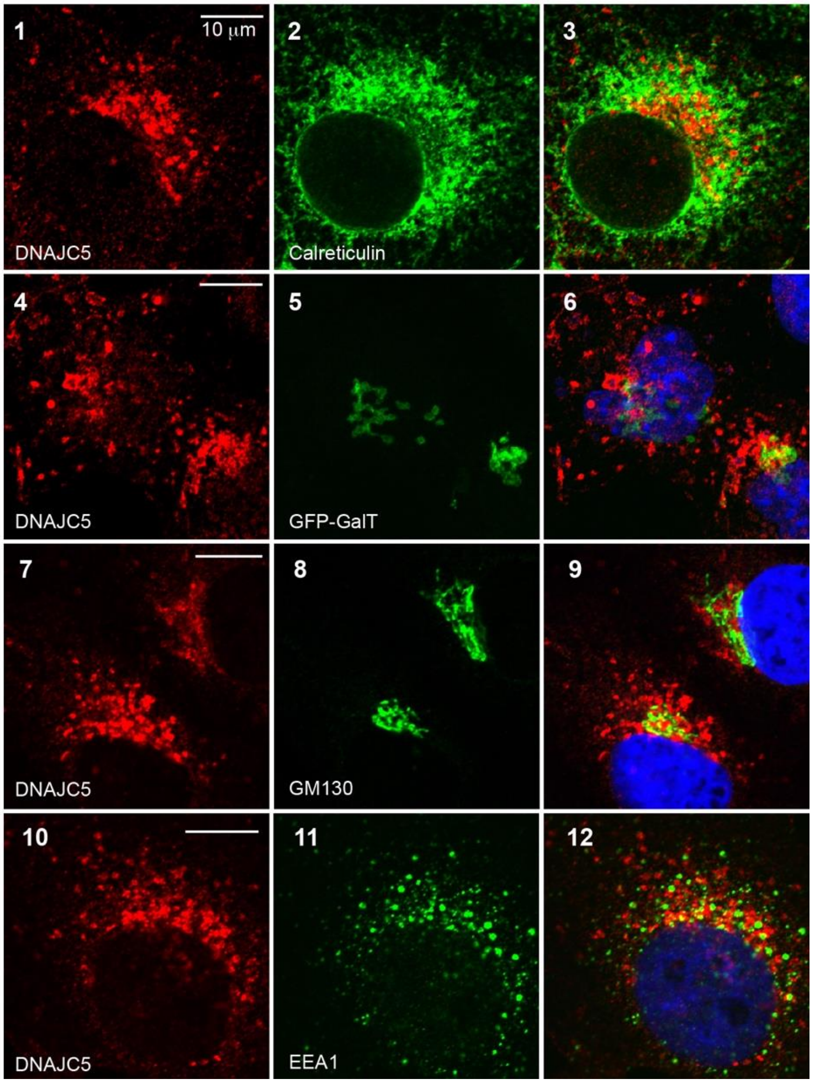
1
10 μm
2
3
DNAJC5
Calreticulin
4
5
6
DNAJC5
GFP-GalT
7
8
9
DNAJC5
GM130
10
11
12
DNAJC5
EEA1

Figure S6 Localization of endogenous DNAJC5 in cells

A, The specificity of the DNAJC5 antibody. COS7 cells transfected with control or DNAJC5 specific siRNA were stained with an antibody against DNAJC5 (red) and protein disulfide isomerase (PDI, green). The nuclei were stained by DAPI in blue. The arrows indicate perinuclear staining of DNAJC5 in control cells. **B**, COS7 cells were fixed and stained with the indicated antibodies. In the case of panels 4-6, cells transfected with the Golgi marker GFP-GalT were used.

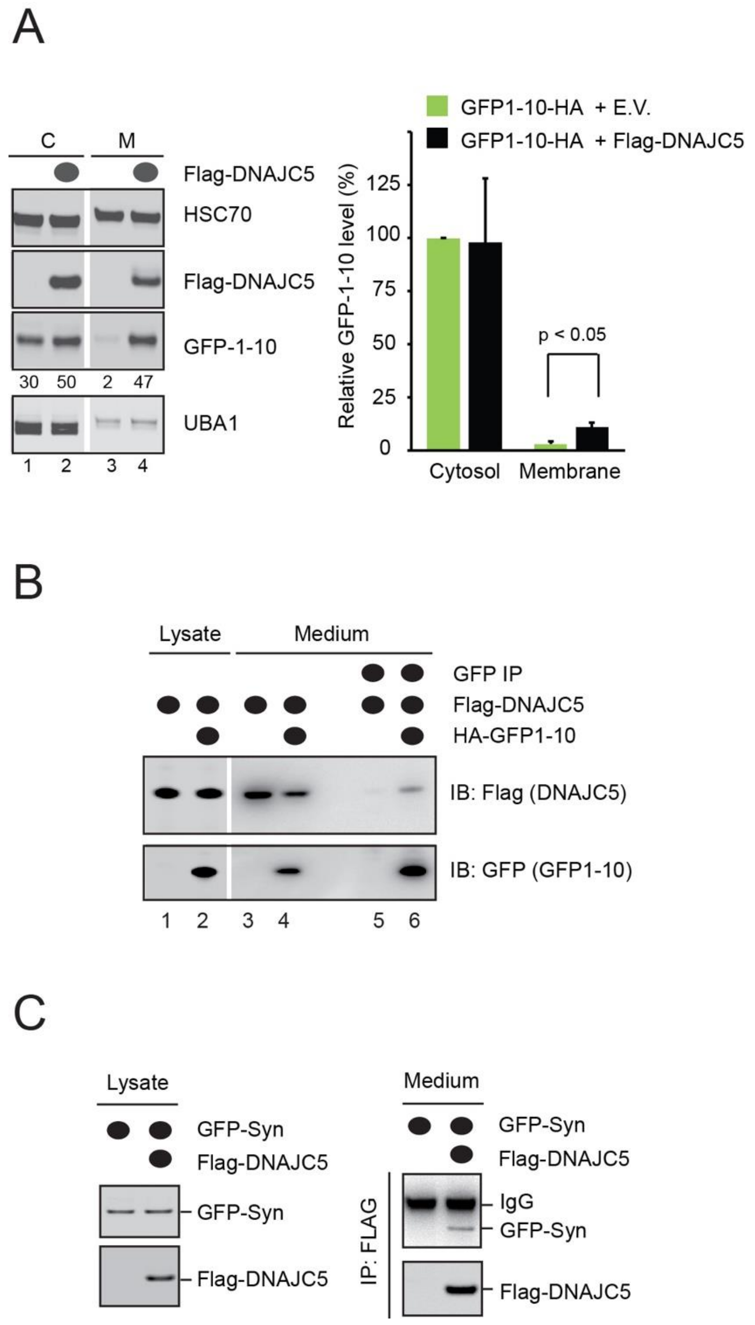

Figure S7 DNAJC5 is co-secreted with MAPS substrates

A, DNAJC5 overexpression promotes membrane recruitment of a MAPS substrate. 293T cells transfected with GFP1-10-HA together with an empty vector (E.V.) or F-DNAJC5 were subject to fractionation and immunoblotting analyses (mean ±s.e.m., n=3). **B**, DNAJC5 interacts with secreted GFP1-10 in conditioned medium. Conditioned medium from cells transfected as indicated was subject to immunoprecipitation (IP) with GFP antibody before immunoblotting (Lanes 5, 6). A fraction of the medium (lanes 3, 4) or cell lysate (lanes 1, 2) was directly analyzed by immunoblotting. **C**, DNAJC5 interacts with secreted α-Syn in conditioned medium.

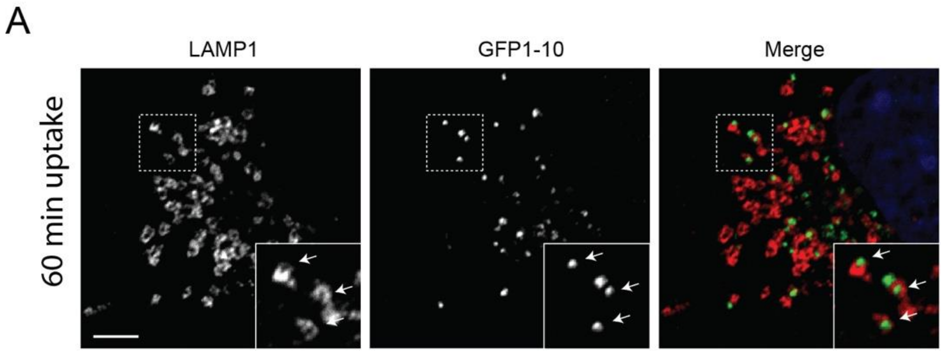

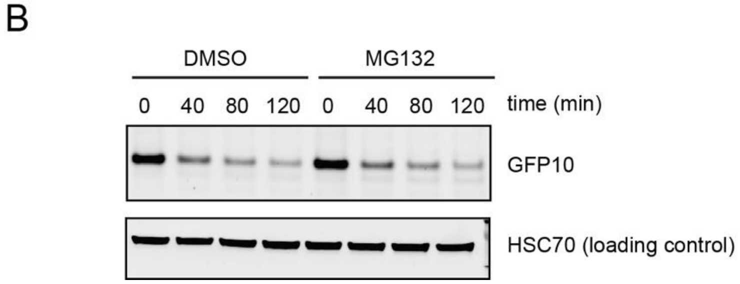

Figure S8 Internalized GFP1-10 can be degraded by the lysosomes

A, Recombinant GFP1-10 was incubated with COS7 cells for 60min at 37 °C in the presence of a protease inhibitor to allow internalization, but not lysosomal degradation. Cells were fixed and stained with antibodies against LAMP1 (red) and GFP (green). **B**, GFP1-10 bound to cells is not degraded by the proteasome. GFP1-10 was incubated with 293T cells to allow surface binding. After removal of unbound protein, cells were incubated in fresh medium in the absence or presence of MG132 (20µM). Cells harvested at the indicated time points were analyzed by immunoblotting.

Supplemental video 1

PA-GFP-DNAJC5 is co-localized with Rab9. An example of COS7 cells expressing pA-GFP-DNAJC5 together with mCherry-Rab9. The cell was treated with a 405nm laser in a peri-nuclear region where Rab9-positive vesicles are concentrated to activate the green fluorescence of DNAJC5.

Supplemental video 2

PA-GFP-DNAJC5 is co-localized with a MAPS substrate. Shown is an example of cells co-expressing pA-GFP-DNAJC5 and mCherry-GFP1-10. After selective photobleaching to remove the mCherry signal from the cytosol (avoiding the peri-nuclear region), the fluorescence of pA-GFP-DNAJC5 was activated in the complementary peri-nuclear region (Green). The cell was then imaged by a LSM780 confocal microscope.

Supplemental video 3

Internalization of EGFP-α-synuclein by COS7 cell. An example of mCherry-Rab5 cells treated for 2 h with conditioned medium harvested from EGFP-α-Synuclein cells transfected with Flag-DNAJC5.

References

1. Lee JG, Takahama S, Zhang G, Tomarev SI, Ye Y. Unconventional secretion of misfolded proteins promotes adaptation to proteasome dysfunction in mammalian cells. *Nat Cell Biol* 2016; **18**: 765-776.
2. Fontaine SN, Zheng D, Sabbagh JJ et al. DnaJ/Hsc70 chaperone complexes control the extracellular release of neurodegenerative-associated proteins. *EMBO J* 2016; **35**: 1537-1549.
3. Bence NF, Sampat RM, Kopito RR. Impairment of the ubiquitin-proteasome system by protein aggregation. *Science* 2001; **292**: 1552-1555.
4. Wang Q, Li L, Ye Y. Regulation of retrotranslocation by p97-associated deubiquitinating enzyme ataxin-3. *J Cell Biol* 2006; **174**: 963-971.
